# Protective effects of statins on COVID-19 risk, severity and fatal outcome: a nationwide Swedish cohort study

**DOI:** 10.1038/s41598-022-16357-2

**Published:** 2022-07-14

**Authors:** Ailiana Santosa, Stefan Franzén, Jonatan Nåtman, Björn Wettermark, Ingela Parmryd, Fredrik Nyberg

**Affiliations:** 1 School of Public Health and Community Medicine, Institute of Medicine, Sahlgrenska Academy, University of Gothenburg, Göteborg, Sweden; 2 National Diabetes Register, Centre of Registers Västra Götaland, Göteborg, Sweden; 3 Pharmacoepidemiology and Social Pharmacy, Department of Pharmacy, Uppsala University, Uppsala, Sweden; 4 Department of Medical Chemistry and Cell Biology, Institute of Biomedicine, Sahlgrenska Academy, University of Gothenburg, Göteborg, Sweden

**Keywords:** Epidemiology, Risk factors

## Abstract

The impact of statins on COVID-19 remains unclear. This study aims to investigate whether statin exposure assessed both in the population and in well-defined cohorts of COVID-19 patients may affect the risk and severity of COVID-19 using nationwide Swedish population-based register data. A population ≥ 40 years was selected by age/sex-stratified random sampling from the Swedish population on 1 Jan 2020. COVID-19 outcomes were identified from the SmiNet database, the National Patient Register and/or Cause-of-Death Register and linked with the National Prescribed Drug Register and sociodemographic registers. Statin exposure was defined as any statin prescriptions in the year before index date. In Cox regressions, confounding was addressed using propensity score ATT (Average Treatment effect in the Treated) weighting. Of 572,695 individuals in the overall cohort, 22.3% had prior statin treatment. After ATT weighting, protective effects were observed among statin user for hospitalization and COVID-19 death in the overall cohort and onset cohort. In the hospitalized cohort, statin use was only associated with lower risk for death (HR = 0.86, 95% CI 0.79–0.95), but not ICU admission. Statin-treated individuals appear to have lower COVID-19 mortality than nonusers, whether assessed in the general population, from COVID-19 onset or from hospitalization.

## Introduction

The COVID-19 pandemic caused by the novel coronavirus SARS-CoV-2 has affected nearly 432 million people worldwide and has led to over 5.9 million confirmed COVID-19-related deaths^1,2^. Various risk factors for severe forms of COVID-19 infection have been identified, including older age, male sex, hypertension, obesity and comorbidities such as cardiovascular disease, diabetes mellitus, and hypertension^3,4^. The 2019 ESC/EAS guidelines for managing dyslipidemia recommend the use of statins for patients with established cardiovascular disease to mitigate overall cardiovascular disease risk^5^. The main mechanism by which statins reduce cardiovascular disease risk is to lower the blood cholesterol level, primarily low-density lipoprotein (LDL), but there are also several important pleiotropic mechanisms such as anti-inflammatory effects, reduction of reactive oxygen species and platelet activity, and lipid regulatory effects that may be important in patients with cardiovascular disease^6^. Some of these may represent mechanisms for modulating the host response among COVID-19 patients. Statins could confer protective effects among COVID-19 patients by stabilizing and promoting regress of atherosclerotic plaques along with anti-inflammatory effects, inhibitor effects on leucocyte–endothelial interaction and inflammatory gene transcription^7^. Additional potential statin mechanisms for a beneficial effect on COVID-19 include plasma membrane effects. Since statins inhibit cholesterol biosynthesis, they could influence the organization of plasma membrane lipid rafts on SARS-CoV-2 target cells in a way that decreases virus adsorption and/or egress of virus particles from infected cells, analogously to the effect statins have on HIV infection^8^, thus reducing the severity of COVID-19 infection. A potential concern with statins and COVID-19 is that statins may increase the expression of ACE2^9^—the membrane receptor that allows SARS-CoV-2 to gain entry into host cells, particularly in lung, heart, vasculature, brain, gastrointestinal tract and kidney—thus suggesting that statins could instead predispose to severe infection and adverse outcomes among COVID-19 patients^10^. On the other hand, ACE2 has both lung- and cardio-protective roles indicating that an increase in its expression may actually ameliorate the COVID-19 outcome^11,12^.

To date, published studies show heterogeneous findings concerning statin use and COVID-19 disease. A meta-analysis based on a few early studies initially reported significant risk reduction for fatal or severe COVID-19 with statins (hazard ratio [HR] = 0.70; 95% confidence interval [CI] 0.53–0.94)^7^. Subsequently, more studies have been conducted, and meta-analyses performed^7,13–18^. Although recent meta-analyses have estimated both no pooled effect of statins^7^ and a protective effect^13^, based on different selections of studies, two carefully conducted meta-analyses noted that pooling of results from studies with adjustment for confounders showed more protective results (pooled OR 0.51 and OR 0.73, respectively), whereas pooling crude estimates did not^15,16^. Overall, many of the studies on statins and COVID-19 were small and with limited or no adjustment for confounders. A majority were based on convenience samples of COVID-19 patients, often hospitalized cohorts, with unclear selection mechanisms—especially those in the earlier phases of the pandemic. The timing of statin exposure was often not clearly assessed. Non-severe outcomes have more rarely been studied concerning statins, but alleviation of COVID-19 symptoms has been reported^19^. The effects of statins on outcomes in patients with COVID-19 thus remain to a large extent unclear^13,14^ and deficiencies in many retrospective studies such as differential handling of comorbidities and confounders and selection issues.

The current study uses a large linked Swedish population-based register database to investigate whether statin exposure both in the general population and in well-defined cohorts of COVID-19 patients may affect multiple outcomes, including the risk, severity and mortality in the COVID-19 study, taking into account comorbidities and confounding factors.

## Methods

### Study population and data sources

This study used data from the SCIFI-PEARL (Swedish Covid-19 Investigation for Future Insights—a Population Epidemiology Approach using Register Linkage) project, described in detail elsewhere^20^. For the current study, a cohort study population including individuals aged 40 and above, free of COVID-19, was obtained by age-sex stratified random sampling of approximately 10% of the Swedish national population on 1 Jan 2020.

We defined 3 study cohorts, with different index dates: (1) overall cohort of all study subjects, with index date 1 Jan 2020; (2) onset cohort of subjects with COVID-19 onset, with index date defined as 2 days prior to the earliest of first positive SARS-CoV-2 polymerase chain reaction (PCR) test results from SmiNet (the national database of notifiable diseases) or COVID-19 International Classification of Diseases, version 10, Swedish edition (ICD-10-SE) code (U07.1 or U07.2) in a healthcare encounter (hospitalization and specialized care visit encounters from the National Patient Register) in our data; (3) hospitalized cohort of all subjects hospitalized for COVID-19, with index date at the admission date. We obtained information on pre-index drug use from the National Prescribed Drug Register, sociodemographic data from Statistics Sweden and comorbidities from the National Patient Register. All information was linked to study subjects using unique personal identifiers.

### Exposure, outcome and follow-up

The main exposure was statin use (ATC code C10AA) in the year prior to index date with the unexposed group including those without any prescription for statin in this time period. In addition, in a sensitivity analysis we also identified statin user based on at least three filled prescriptions in the year before the index date. Prescriptions for chronic drug use are generally dispensed for 3-month periods (or 100 days, due to pack sizes) each time prescriptions are filled in Sweden. Individuals were grouped as those who regularly used (exposed) and those who did not regularly use statins (unexposed) according to this definition.

Follow-up for each cohort started on the day after index date and extended for each subject to the earliest of outcome, death, emigration or end of follow-up on 20 Jan 2021. The outcomes investigated were:

1. *COVID-19 test-positivity*: Individuals with positive PCR test result for SARS-CoV-2 in SmiNet. Event date was the test sampling date.
2. *COVID-19 diagnosis*: Individuals with healthcare encounter with COVID-19 ICD-10 code in the Patient Register or the Cause-of-Death register, or positive test result for SARS-CoV-2 in SmiNet. Event date was defined as the earliest of these.
3. *COVID-19 hospitalization*: Individuals admitted to hospital based on primary or secondary diagnosis with COVID-19 ICD-10 code in the Patient Register. Event date was the date of hospital admission.
4. *COVID-19 ICU admission*: Individuals who were transferred to or were directly admitted in the Intensive Care Unit (ICU) with COVID-19 diagnosis based on the Swedish Intensive Care Register. Event date was the date of ICU admission.
5. *COVID-19 mortality*: Individuals identified from Cause-of-death register with COVID-19 ICD-10 code as the underlying or contributing cause of death. Event date was the date of death.

### Statistical analysis and adjustment covariates

Descriptive statistics are presented as means with standard deviation for continuous variables and counts with percentage for categorical variables. We calculated standardized mean difference (SMD) to evaluate the balance between treated and untreated groups before and after propensity score weighting. The unadjusted number of events and event rates (per 1000 person-years) with 95% exact Poisson Confidence Intervals (CI) for COVID-19 endpoints were computed for statin users and non-users. The cumulative incidence of COVID-19 endpoints was estimated as one minus the Kaplan–Meier estimate of the survival function and are presented with 95% CIs.

In the primary analyses, Cox regressions with statin exposure as the sole independent variable were used. We computed hazard ratios (HRs) unadjusted and adjusted using propensity scores for weighting to estimate the average treatment effect for the treated (ATT) using ATT weights, also known as standardized mortality ratio (SMR) weights^21^. The propensity scores were estimated using gradient boosting (twang package in R) with shrinkage set to 0.01, interaction depth 3^22^. The number of trees was optimized to give optimal weighted balance between the treatment groups as determined by minimizing the average weighted SMD across the variables in the propensity score model. The ATT weight-adjusted Cox proportional hazards regression model used robust standard errors to account for the weights. The models estimated HR with 95% CI. p values below 0.05 were considered statistically significant, with no adjustment for multiple comparisons. We used R software (version 4.0.2) for statistical analyses. We also stratified the analysis by sex and performed a sensitivity analysis using a different exposure definition, as described above.

Covariates in the propensity score included age, sex, education, marital status, employment, disposable income, country of birth, comorbidities **(**hypertension, cardiovascular disease, ischemic heart disease, stroke or transient ischemic attack (TIA), arrhythmia, heart failure, diabetes mellitus, chronic kidney disease, respiratory disease, cancer, liver disease, obesity, neurological disease, dementia, and autoimmune disease) and prior medications (including angiotension converting enzyme inhibitors (ACEI), angiotension receptor blockers (ARB), calcium channel blockers (CCB), diuretics, betablockers, ezetimib, novel oral anticoagulants (NOACs), platelet P2Y12 receptor inhibitors, metformin, sulfonylurea, insulin, glucosidase inhibitors, pioglitazone, dipeptidyl peptidase 4 (DPP-4) inhibitors, glucagon-like peptide 1 (GLP-1) receptor agonists, sodium-glucose co-transporter-2 (SGLT2) inhibitors, neuroleptics, and respiratory treatments), see S1 Table and S2 Table for variable definitions and data capture periods for comorbidities and drug exposures). There were very limited missing data for any of the variables (largely per definition as many comorbidities and drugs were defined as present (“yes”) when registered, otherwise “no” when absent), and where present, missingness was incorporated in the gradient boosting propensity score algorithm^22^.

Ethical approval for this research was obtained from the Swedish Ethical Review Authority (EPM), no. 2020-01800, 2020-05829, 2021-00267, 2021-00829, 2021-02106, 2021-04098. All study procedures involving human participants followed the ethical standards of the institutional research committee, as well as the 1964 Helsinki Declaration and its amendments, or equivalent ethical standards. In Sweden, informed consent is generally not required for large-scale, registry-based studies. In this study, the Ethical Committee did not require the research team to collect informed consent from all individuals. Additionally, all linked information has been anonymized and de-identified by the National Board of Health and Welfare and Statistics Sweden, respectively, before reaching University of Gothenberg and the research team.

## Results

Among 572,695 individuals aged ≥ 40 years in the overall population cohort, 22.3% (n = 127,542) were statin users (Table 1), and 15.2% (n = 79,769) (S3 Table) were regular statin users as defined for the sensitivity analysis. The mean age of statin users (75.9 ± 11.7 years) was higher than nonusers (65.9 ± 17). Statin users were more likely to have low education (35.4% vs 23.9%), be not gainfully employed/retired (71.8% vs 45.8%), and have low income, as well as more comorbidities and other medications (Table 1). Corresponding descriptive data for the COVID-19 onset and hospitalized cohorts are given in the S4 Table and S5 Table. The propensity scores among treated and untreated for the three cohorts had well overlapping distributions before weighting (S1 Figure). After propensity score weighting, a good balance was achieved between the untreated and treated groups, with all SMDs below 0.082 in all study cohorts (Table 1, S4 Table, S5 Table).

**Table 1:** Demographic and socioeconomic characteristics, comorbidities and prior medication of the *overall population cohort* on 1 Jan 2020 with standardized mean differences (SMDs) before and after ATT weighting, by statin use status (presented as n (%)).

Characteristics	Statin users	Nonusers	SMD (before ATT weighting)	SMD (after ATT weighting)
N	127 542	445 153		
Women	52,798 (41.4%)	239,592 (53.8%)	0.251	0.004
Age, years (mean ± SD)	75.9 ± 11.7	65.9 ± 17.0	0.686	0.019
**Age group (years)**				
40–49	2932 (2.3%)	97,067 (21.8%)	0.891	0.019
50–59	9661 (7.6%)	90,339 (20.3%)		
60–69	23,067 (18.1%)	76,931 (17.3%)		
70–79	36,927 (29.0%)	63,072 (14.2%)		
80–89	39,018 (30.6%)	60,973 (13.7%)		
90 +	15,937 (12.5%)	56,771 (12.8%)		
**Education level**				
Low (primary school)	44,516 (35.4%)	104,620 (23.9%)	0.304	0.005
Medium (secondary school)	52,586 (41.8%)	182,935 (41.8%)		
High (postgraduate)	28,763 (22.9%)	150,305 (34.3%)		
Unemployed	91,603 (71.8%)	203,716 (45.8%)	0.549	0.001
Married	62,205 (48.8%)	206,803 (46.5%)	0.046	0.001
Disposable income, SEK (mean ± SD)	2516.7 ± 7452.5	2951.8 ± 6653.4	0.062	0.014
**Country of birth**				
Sweden	108,261 (84.9%)	366,656 (82.4%)	0.127	0.028
Nordic	6151 (4.8%)	16,110 (3.6%)		
EU	3267 (2.6%)	13,630 (3.1%)		
Rest of the world	9863 (7.7%)	48,757 (11.0%)		
**Comorbidities**				
Hypertension	57,012 (44.7%)	69,967 (15.7%)	0.665	0.005
Cardiovascular disease	50,426 (39.5%)	59,543 (13.4%)	0.621	0.011
Ischemic heart disease	31,418 (24.6%)	14,762 (3.3%)	0.646	0.016
Stroke or TIA	15,439 (12.1%)	9865 (2.2%)	0.391	0.002
Arrhythmia	24,408 (19.1%)	37,471 (8.4%)	0.315	0.007
Heart failure	13,763 (10.8%)	18,063 (4.1%)	0.259	0.007
Diabetes	26,940 (21.1%)	16,186 (3.6%)	0.551	0.006
Chronic kidney disease	6423 (5.0%)	6920 (1.6%)	0.196	0.001
Respiratory disease	6214 (4.9%)	9023 (2.0%)	0.156	0.001
Cancer	21,373 (16.8%)	45,436 (10.2%)	0.193	0.002
Liver disease	793 (0.6%)	2249 (0.5%)	0.016	0.005
Obesity	3144 (2.5%)	5125 (1.2%)	0.099	0.002
Neurological disease	2247 (1.8%)	6036 (1.4%)	0.033	< 0.001
Dementia	3789 (3.0%)	11,155 (2.5%)	0.028	0.003
Autoimmune	8279 (6.5%)	17,168 (3.9%)	0.119	0.005
**Prior medications**				
ACEI	40,571 (31.8%)	44,459 (10.0%)	0.557	0.005
ARB	44,838 (35.2%)	60,288 (13.5%)	0.520	0.006
CCB	46,725 (36.6%)	61,604 (13.8%)	0.544	0.008
Diuretics	41,760 (32.7%)	70,366 (15.8%)	0.403	0.001
Betablockers	67,020 (52.5%)	77,218 (17.3%)	0.794	0.018
Ezetimib	5273 (4.1%)	1859 (0.4%)	0.251	0.014
Other lipid lowering	731 (0.6%)	1565 (0.4%)	0.033	0.002
Insulin	14,668 (11.5%)	7961 (1.8%)	0.398	0.007
Metformin	26,895 (21.1%)	12,655 (2.8%)	0.586	0.001
Sulfonylureas	3216 (2.5%)	1574 (0.4%)	0.183	0.004
Glucosidase inhibitors	64 (0.1%)	49 (0.0%)	0.022	0.012
Pioglitazone	357 (0.3%)	142 (0.0%)	0.063	0.004
DPP4 inhibitors	7589 (6.0%)	3437 (0.8%)	0.290	0.011
GLP1 agonists	3550 (2.8%)	1247 (0.3%)	0.205	0.007
SGLT2 inhibitors	4575 (3.6%)	1303 (0.3%)	0.241	0.010
Neuroleptics	3046 (2.4%)	11,654 (2.6%)	0.015	0.042
Anti-depressants	23,358 (18.3%)	66,880 (15.0%)	0.088	0.001
Anxiolytics	12,842 (10.1%)	39,130 (8.8%)	0.044	0.001
Sedative	26,276 (20.6%)	64,162 (14.4%)	0.163	0.002
P2Y12 inhibitors	14,772 (11.6%)	4439 (1.0%)	0.447	0.004
ASA cardiac	53,188 (41.7%)	32,322 (7.3%)	0.874	0.008
NOACs	18,304 (14.4%)	28,914 (6.5%)	0.259	0.003
Vitamin K antagonists	8912 (7.0%)	11,718 (2.6%)	0.205	< 0.001
Respiratory drugs	18,212 (14.3%)	46,065 (10.3%)	0.120	0.004

*ATT* average treatment effect for the treated, *TIA* transient ischemic attack, *ACEI* angiotension converting enzyme inhibitors, *ARB* angiotension receptor blockers, *CCB* calcium channel blockers, *NOACs* novel oral anticoagulants, *DPP-4* dipeptidyl peptidase 4 inhibitors, *SGLT2* sodium-glucose co-transporter-2.

Table 2 shows the crude incidence rates per 1000 person-years among statin users and nonusers for various COVID-19 outcomes in the three studied cohorts. Whereas the incidence rates for test-positivity and diagnosis of COVID-19 were somewhat lower among statin users compared to non-users in the overall population cohort, incidence rates for hospitalization, ICU admission and death related to COVID-19 were clearly higher among statin users than non-users in both the overall population cohort and the onset cohort. For the hospitalized cohort, the COVID-19 mortality rate was similarly almost twice as high in statin users as in nonusers (609.1/1000 person-years vs. 385.3/1000 person-years), but we did not observe a similar trend for the incidence rate of ICU admission, which was almost identical in statin users and nonusers. In the sensitivity analysis with the exposed group restricted to regular statin users, we observed a similar trend for the three studied cohorts (S6 Table). Cumulative incidence Kaplan–Meier curves for each outcome in the three cohorts illustrate the same observed differences over time and subdivided by sex (S2–S4 Figures). Overall, the difference in cumulative probability for each outcome was greater among men than women.

**Table 2:** Events, person-years of observation, and crude incidence rates per 1000 person-years [with 95% exact Poisson confidence intervals (CI)] for various COVID-19 outcomes in the three studied cohorts (overall population cohort, COVID-19 onset cohort and hospitalized cohort), by statin use.

Cohort	COVID-19 outcomes	Statin users			Non-users			P value
		Events	Person-years	Incidence rates [95% CI]	Events	Person-years	Incidence rates [95% CI]	
Overall cohort		N = 127,542			N = 445,153			
	Test-positive	5822	128,885	45.2 [44.0–46.3]	25,376	450,753	56.3 [55.6–57.0]	< 0.001
	Diagnosis	6389	128,667	49.7 [48.4–50.9]	26,633	450,297	59.1 [58.4–59.9]	< 0.001
	Hospitalization	2902	129,684	22.4 [21.6–23.2]	6238	454,928	13.7 [13.4–14.1]	< 0.001
	ICU admission	166	130,037	1.30 [1.10–1.50]	264	455,684	0.60 [0.50–0.70]	< 0.001
	Death	1042	130,084	8.0 [7.50–8.50]	2722	455,782	6.0 [5.70–6.20]	< 0.001
Onset cohort		N = 52,128			N = 242,785			
	Hospitalization	15,227	8521	1787.1 [1758.8–1815.7]	24,857	46,129	538.9 [532.2–545.6]	< 0.001
	ICU admission	1543	13,627	113.2 [107.7–119]	2701	54,901	49.2 [47.4–51.1]	< 0.001
	Death	4208	12,596	334.1 [324.0–344.3]	6282	53,522	117.4 [114.5–120.3]	< 0.001
Hospitalized cohort		N = 15,246			N = 24,846			
	ICU admission	1522	5196	292.9 [278.4–308]	2673	8910	300 [288.7–311.6]	< 0.001
	Death	2884	4735	609.1 [587.1–631.8]	3386	8788	385.3 [372.4–398.5]	< 0.001

For the association analyses, unadjusted and fully ATT weight-adjusted HRs for statin use in the three studied cohorts for the 5 outcomes are shown in Table 3. With ATT weight adjustment to remove confounding, hazard ratios for the overall population cohort changed considerably, so that statin users had a significantly lower risk for test-positivity, diagnosis, hospitalization and death related to COVID-19 (p < 0.05), but not for ICU admission (Table 3). In the onset cohort, reduced risk were seen after ATT weight adjustment for all outcomes of hospitalization, ICU admission and death (Table 3). In the hospitalized population cohort on the other hand, after ATT weight adjustment, prior statin exposure was significantly associated with lower risk only for COVID-19 death (HR 0.86, 95% CI 0.79–0.95), but no clear evidence of association for prior statin exposure with COVID-19-related ICU admission was seen (Table 3).

**Table 3:** Hazard ratios (HRs), unadjusted and adjusted using ATT weighting, with 95% confidence interval (CI), comparing the risk of various COVID-19 outcomes between statin users and non-users, in the 3 studied cohorts (overall population cohort, COVID-19 onset cohort and hospitalized cohort).

	Unadjusted HR						ATT weight-adjusted HR					
	Total		Men		Women		Total		Men		Women	
	HR (95% CI)	p value	HR (95% CI)	p value	HR (95% CI)	p value	HR (95% CI)	p value	HR (95% CI)	p value	HR (95% CI)	p value
**Overall cohort**												
Test-positive	0.80 [0.78–0.83]	< 0.001	0.89 [0.86–0.93]	< 0.001	0.72 [0.69–0.75]	< 0.001	0.90 [0.85–0.95]	< 0.001	0.92 [0.85–0.98]	0.02	0.87 [0.81–0.94]	< 0.001
Diagnosis	0.84 [0.82–0.86]	< 0.001	0.93 [0.90–0.96]	< 0.001	0.76 [0.73–0.79]	< 0.001	0.90 [0.85–0.94]	< 0.001	0.91 [0.85–0.97]	0.007	0.88 [0.82–0.95]	< 0.001
Hospitalization	1.68 [1.60–1.76]	< 0.001	1.69 [1.59–1.79]	< 0.001	1.56 [1.45–1.68]	< 0.001	0.86 [0.78–0.93]	< 0.001	0.85 [0.76–0.94]	0.002	0.87 [0.75–1.01]	0.07
ICU admission	2.2 [1.81–2.67]	< 0.001	1.96 [1.56–2.47]	< 0.001	2.13 [1.46–3.10]	< 0.001	0.99 [0.67–1.46]	0.97	0.99 [0.61–1.60]	0.97	0.99 [0.58–1.70]	0.97
Death	1.33 [1.23–1.42]	< 0.001	1.44 [1.31–1.57]	< 0.001	1.07 [0.95–1.21]	0.053	0.69 [0.62–0.77]	< 0.001	0.68 [0.59–0.77]	< 0.001	0.73 [0.62–0.85]	< 0.001
**Onset cohort**												
Hospitalization	3.21 [3.14–3.27]	< 0.001	2.82 [2.75–2.9]	< 0.001	3.46 [3.35–3.57]	< 0.001	0.93 [0.90–0.97]	< 0.001	0.90 [0.86–0.95]	< 0.001	0.98 [0.92–1.03]	0.39
ICU admission	2.68 [2.52–2.86]	< 0.001	2.22 [2.07–2.39]	< 0.001	2.85 [2.52–3.22]	< 0.001	0.87 [0.77–0.99]	0.04	0.90 [0.77–1.04]	0.16	0.78 [0.62–1.00]	0.05
Death	3.21 [3.09–3.34]	< 0.001	3.26 [3.09–3.43]	< 0.001	2.96 [2.78–3.14]	< 0.001	0.81 [0.76–0.87]	< 0.001	0.83 [0.75–0.91]	< 0.001	0.78 [0.71–0.86]	< 0.001
**Hospitalized cohort**												
ICU admission	0.92 [0.87–0.98]	0.014	0.87 [0.81–0.93]	< 0.001	0.90 [0.79–1.02]	0.09	0.98 [0.87–1.11]	0.81	1.03 [0.89–1.19]	0.70	0.86 [0.69–1.08]	0.19
Death	1.45 [1.38–1.52]	< 0.001	1.52 [1.43–1.62]	< 0.001	1.30 [1.20–1.41]	< 0.001	0.86 [0.79–0.95]	0.001	0.88 [0.78–1.00]	0.04	0.83 [0.73–0.94]	0.004

In the sensitivity analysis, we observed quite similar trends for the hazard ratios for COVID-19 infection, severity and death among regular statin user vs. non-user in all three studied cohorts, with a greater protective effect for the risk of COVID-19 death in the overall cohort population (HR = 0.74, 95% CI 0.66–0.83) (S7 Table).

When we stratified the analyses by sex, after ATT weight adjustment, the hazard ratio for COVID-19 death in the overall population cohort was significantly reduced for both men (0.68, 95% CI 0.59–0.77) and women (0.73, 95% CI 0.62–0.85) with prior statin exposure (Table 3). Although across all comparison there was some suggestion of stronger associations in women, differences between the sexes were not strong or consistent. In the sensitivity analysis, very similar stratified results were seen (S7 Table).

## Discussion

This study investigated the impact of prior statin treatment on a range of five different COVID-19 outcomes in three different Swedish population-based cohorts—general population, individuals with COVID-19 onset and a COVID-19 hospitalization cohort. Our main findings show that prior statin treatment was significantly associated with a reduced risk of COVID-19 test-positivity, diagnosis, hospitalization and mortality in the 3 studied population cohorts. These findings strengthen and support evidence on the hypothesis of pleiotropic protective effects of statins in COVID-19 from prior cohort studies^23–30^ and align with evidence summarized by recent meta-analyses and systematic reviews on the association of statin use and the potential protective effects against progression and severity of COVID-19^15,16,18,31,32^. A recent Swedish cohort study using register data with a smaller sample size limited to the Stockholm Region similarly showed statin use to be a protective factor for COVID-19 death^29^. That study had shorter follow-up than ours, but similar to our main exposure definition, defined statin exposure broadly as any statin treatment initiated before the pandemic, not regular statin use).

As noted previously, plausible biological and clinical mechanisms, both direct and indirect, by which statins might protect against COVID-19 disease or severity have been proposed^7,15,16,33–35^. Statins have potential effects to reduce the cytokine release syndrome in COVID-19 by inhibiting Toll-like receptor 4 (TLR4) and down-modulating macrophage activity^36–38^. Statins have further been demonstrated to suppress the expression of both TLR2 and TLR4, leading to an immune response shift towards an anti-inflammatory response^39^.

Beyond their cholesterol-lowering effect, statins could also affect the plasma membrane composition, where cholesterol is a dominating component. This effect is particularly important for enveloped viruses like SARS-CoV-2 that must pass the plasma membrane twice upon cell infection and again upon egress of newly synthesized virus particles. Recent studies have demonstrated that cholesterol is important in forming syncytia, multinucleated cells, that characterizes SARS-CoV-2 cell entry^40^. Statins may well cause a reduction of syncytia by reducing plasma membrane cholesterol levels, thus reducing viral infection. Preliminary results indicate that statins indeed can reduce SARS-CoV-2 infection of lung epitheial cells (I. Parmryd, personal communication).

Statins also exert pleiotropic effects affecting inflammation and oxidative stress^36^ of potential relevance for infection and lung protection. Statins may diminish the complications of COVID-19 by improving endothelial function, reducing serum PAI-1 levels and attenuating TGF-and VEGF in lung tissue^34^. Furthermore, statins can constrain SARS-CoV-2 reproduction by restraining the main protease (Mpro) and RNA-dependent RNA polymerase (RdRp)^34^. Moreover, the effectiveness of statin treatment in significantly decreasing hospitalizations and deaths has been shown for influenza and Ebola virus diseases^41,42^.

Alongside their potential benefits in COVID-19, the side effects of statin treatment need to be considered, such as elevated creatinine kinase (CK) and elevated serum glucose levels, which have been reported in severe COVID-19 patients^43^. Although statin treatment is commonly considered safe and well-tolerated, statins may induce liver injury^44^. Current European guidance for the diagnoses and management of cardiovascular disease during the COVID-19 pandemic recommends temporarily restraining statin treatment in patients with high liver enzymes^45^. Future studies should preferably assess both positive and negative statin effects in COVID-19 patients.

Conflicting findings of the beneficial effects of prior statin treatment on COVID-19 have been reported in retrospective studies. Recent cohort studies have reported increased, decreased and unaffected risk for COVID-19 severity and mortality^46–50^. Residual confounding may be an issue, and more carefully adjusted studies show more consistent protective results^15,16^. We have investigated individuals at three stages in the COVID-19 disease progression using 3 different cohorts: healthy before disease onset, from COVID-19 onset, and from COVID-19 hospitalization—with careful adjustment for confounding using propensity score analysis with ATT weighting. Protective effects of statins were seen for COVID-19 death for all three cohorts and in our overall population cohort and COVID-19 onset population also for the less severe outcomes (hospitalization), supporting a protective effect of statin on COVID-19 infection and severity. Despite some indications of an overall stronger protection in women than in men, we did not really observe consistent sex differences, which would have been consistent with the overall higher risk for severe COVID-19 outcomes seen in men during the pandemic^51^.

Despite current guidelines recommending continued statin treatment in patients who were previously taking these drugs because of the known effects of statins on ACE2, statin treatment may be discontinued in patients with COVID-19. In addition, guidelines suggest an individualized approach in deciding prescribing statins to older people^52^, which may include considering discontinuation, especially in cases of severe illness and frailty. A hospital-based study in the US showed that the odds of mortality decreased by 35% in statin-users who continued to use statins upon hospital admission for COVID-19 compared with those for which the. statin therapy was discontinued^53^. The effect of discontinuation of statin treatment on the risk of COVID-19 in a population needs to be investigated in further detail.

Our study has several strengths and limitations that merit discussion. Using a population-based database in Sweden, we investigated the impact of statin use both in the population and in well-defined cohorts of COVID-19 patients for several different clinical COVID-19 outcomes. The completeness of data from nationwide administrative registries (including high-quality socio-economic data) and efficient linkages of different register data for COVID-19 patients to identify test-positive disease, clinical diagnoses and severe COVID-19 outcomes (hospitalization, ICU admission and death) are fundamental strengths of this study and support generalization of the results. In this study, we outlined statin exposure in the prior year into two different ways. Our main exposure definition was any statin exposure in the prior year, consistent with definitions commonly used in many studies. As a sensitivity analysis, we also used a more specific exposure definition, regular statin use, defined as ≥ 3 (generally 3-month) prescriptions in the prior year, to better capture individuals with likely ongoing regular statin use at index date, recognizing the variable adherence to actual daily statin intake in real-life data. Our sensitivity analysis with this definition gave very similar results, suggesting that the main exposure definition has adequate specificity for the hypothesized true exposure. We assume that short-term mechanisms of current use at the index would most likely be relevant for the observed effects we report. However, the study also has some limitations. Some exposure misclassification is possible, both of statin exposed and unexposed individuals, but this is unlikely to be related to the outcomes (which were totally unanticipated prior to index dates), and potential bias would thus tend to be towards the null. Unless the exposure misclassification is substantial, and the effect in the misclassified individuals is highly different, such bias would also have limited impact. Misclassification errors in identifying COVID-19 outcomes are also possible, especially under ascertainment of test-positive milder disease, but it is unlikely this differs between statin-treated and untreated. For the more severe outcomes, this issue is likely of less concern. Although propensity score weighting was used to minimize potential confounding, we cannot exclude some remaining bias from patient selection, treatment indication and residual confounding due to unmeasured confounders such as health risk behaviour (physical activity, smoking) and laboratory parameters (dyslipidemia). Nevertheless, the exposure cohorts were quite well balanced after propensity score weighting, and the results were consistent across the studied cohorts and follow-up periods from different index time points.

## Conclusions

Findings from this study provide broad evidence of a beneficial effect of prior statin exposure on COVID-19 mortality in all the three cohort populations studied (overall population, COVID-19 onset and COVID-19 hospitalization cohorts), as well as less strong effects on other COVID-19 outcomes along the COVID-19 progression spectrum of outcomes that we studied. Together with prior available scientific evidence, both mechanistic and epidemiological, our findings support potential investigation into repurposing of statins for COVID-19 protection.

## Supplementary Information

Supplementary Information.

## Data Availability

The data in this study are pseudonymized individual-level data from Swedish healthcare registers and are not publicly available according to Swedish legislation. They can be obtained from the respective Swedish public data holders on the basis of ethics approval for the research in question, subject to relevant legislation, processes and data protection.
