# Supplementary material for: Protective effects of statins on COVID-19 risk, severity and fatal outcome: a nationwide Swedish cohort study
Source: Sci Rep. 2022 Jul 14;12:12047. doi: 10.1038/s41598-022-16357-2 (PMC9282150; doi:10.1038/s41598-022-16357-2)
Supplement: Supplementary file 1 — Supplementary Information. [file 41598_2022_16357_MOESM1_ESM.docx]

# Supplementary material

This supplementary material has been provided by the authors to give readers additional information about their work.

Supplementary material to: Protective effects of statins on COVID-19 risk, severity and fatal outcome – a nationwide Swedish cohort study

**S1 Table. ICD-10 codes for prior comorbidities, assessed in the five years prior to index date**

| **Comorbidity** | **ICD code** |
| --- | --- |
| Hypertension | I10-I15 |
| Cardiovascular disease | I05-I09 I20-I51 R001 R011 Q20-Q28 |
| Ischemic heart disease | I20 I21 I22 I23 I24 I25 |
| Stroke or TIA | I61 I62 I63 I64 G45 |
| All arrhythmias | I441 I442 I452 I453 I456 I459 I46 I47 I48 I490 I495 |
| Heart failure | I50 |
| Diabetes | E10 E11 E13 E14 |
| Chronic Kidney Disease | N18 |
| Respiratory disease | J439 J431 J432 J438 J430 J448 J449 J849 J841 J848 J840 J961 J969 E840 E849 E848 E841 |
| Cancer | C00-C97 |
| Liver disease | K70-K77 |
| Obesity | E66 |
| Neurological disease | G20 G35 G12 |
| Dementia | F00 F01 F02 F03 |
| Autoimmune disease | M05-M14 |

**S2 Table. ATC codes for prior medications, assessed in the year prior to index date**

| **Drug** | **ATC code** |
| --- | --- |
| ACEI | C09A |
| ARB | C09C |
| CCB | C08 |
| Diuretics | C03 |
| Betablockers | C07 (except C07AA07) |
| Ezetimib | C10AX09 |
| Other lipid lowering | C10AB |
| Insulin | A10A |
| Metformin | A10BA02 A10BD05 A10BD07  A10BD08 A10BD10 A10BD11 A10BD15 A10BD20 A10BD23 |
| Sulfonylureas | A10BB |
| Glucosidase inhibitors | A10BF |
| Pioglitazone | A10BG03 |
| DPP4 inhibitors | A10BH A10BD07 A10BD08 A10BD10  A10BD11 A10BD19 A10BD21 A10BD24 |
| GLP1 receptor agonists | A10BJ |
| SGLT2 inhibitors | A10BK A10BD15 A10BD20 A10BD23 A10BD19  A10BD21 A10BD24 |
| Neuroleptics | N05A |
| Anti-depressants | N06A |
| Anxiolytics | N05B |
| Sedative | N05C |
| P2Y12 receptor inhibitors | B01AC04 B01AC22 B01AC24 B01AC25 |
| ASA cardiac | B01AC06 |
| NOACs | B01AE B01AF |
| Vitamin K antagonists | B01AA |
| Respiratory drugs | R03A R03BA R03BB R03C R03D |

**S3 Table.** Demographic and socioeconomic characteristics, comorbidities and prior medication of the *overall population cohort* on their index date, with standardized mean differences (SMD) before and after ATT weighting, by regular statin use status (presented as n (%))

| **Characteristics** | | **Statin users** | **Nonusers** | **SMD**  **(before ATT weighting)** | **SMD**  **(after ATT weighting)** |
| --- | --- | --- | --- | --- | --- |
| N | | 79769 | 445153 |  |  |
| Women | | 32799 (41.1) | 239592 (53.8) | 0.257 | 0.003 |
| Age, years (mean±SD) | | 76.74 (11.36) | 65.85 (17.02) | 0.752 | 0.026 |
| Age group (years) | | | | | |
|  | 40-49 | 1395 ( 1.7) | 97067 (21.8) | 0.948 | 0.017 |
|  | 50-59 | 5207 ( 6.5) | 90339 (20.3) |  |  |
|  | 60-69 | 13562 (17.0) | 76931 (17.3) |  |  |
|  | 70-79 | 22985 (28.8) | 63072 (14.2) |  |  |
|  | 80-89 | 25754 (32.3) | 60973 (13.7) |  |  |
|  | 90+ | 10866 (13.6) | 56771 (12.8) |  |  |
| Education level | | | | | |
|  | Low (Primary school) | 28981 (36.8) | 104620 (23.9) | 0.337 | 0.003 |
|  | Medium (Secondary school) | 32586 (41.4) | 182935 (41.8) |  |  |
|  | High (Postgraduate) | 17183 (21.8) | 150305 (34.3) |  |  |
| Unemployed | | 59102 (74.1) | 203716 (45.8) | 0.604 | 0.001 |
| Married | | 38128 (47.8) | 206803 (46.5) | 0.027 | 0.001 |
| Disposable income, SEK (Mean±SD) | | 2479.28 (6821.82) | 2951.83 (6653.39) | 0.070 | 0.018 |
| Country of birth | |  | | |  |
|  | Sweden | 68757 (86.2) | 366656 (82.4) | 0.166 | 0.050 |
|  | Nordic | 3810 ( 4.8) | 16110 ( 3.6) |  |  |
|  | EU | 1832 ( 2.3) | 13630 ( 3.1) |  |  |
|  | Rest of the world | 5370 ( 6.7) | 48757 (11.0) |  |  |
| **Comorbidities** | | | | | |
|  | Hypertension | 37054 ( 46.5) | 69967 ( 15.7) | 0.704 | 0.006 |
|  | Cardiovascular disease | 33198 ( 41.6) | 59543 ( 13.4) | 0.667 | 0.010 |
|  | Ischemic heart disease | 20750 ( 26.0) | 14762 ( 3.3) | 0.677 | 0.013 |
|  | Stroke or TIA | 10214 ( 12.8) | 9865 ( 2.2) | 0.410 | 0.002 |
|  | Arrhythmia | 17819 ( 22.3) | 41557 ( 9.3) | 0.362 | 0.009 |
|  | Heart failure | 9418 ( 11.8) | 18063 ( 4.1) | 0.290 | 0.006 |
|  | Diabetes | 17581 ( 22.0) | 16186 ( 3.6) | 0.572 | 0.001 |
|  | Chronic kidney disease | 4282 ( 5.4) | 6920 ( 1.6) | 0.210 | 0.001 |
|  | Respiratory disease | 4128 ( 5.2) | 9023 ( 2.0) | 0.170 | 0.001 |
|  | Cancer | 13668 ( 17.1) | 45436 ( 10.2) | 0.203 | 0.005 |
|  | Liver disease | 453 ( 0.6) | 2249 ( 0.5) | 0.009 | 0.004 |
|  | Obesity | 1976 ( 2.5) | 5125 ( 1.2) | 0.099 | 0.007 |
|  | Neurological disease | 1507 ( 1.9) | 6036 ( 1.4) | 0.042 | 0.004 |
|  | Dementia | 2857 ( 3.6) | 11155 ( 2.5) | 0.063 | 0.002 |
|  | Autoimmune | 5278 ( 6.6) | 17168 ( 3.9) | 0.124 | 0.006 |
| **Prior medications** | | | | | |
|  | ACEI | 26276 ( 32.9) | 44459 ( 10.0) | 0.582 | 0.004 |
|  | ARB | 28014 ( 35.1) | 60288 ( 13.5) | 0.520 | 0.005 |
|  | CCB | 29773 ( 37.3) | 61604 ( 13.8) | 0.559 | 0.010 |
|  | Diuretics | 27499 ( 34.5) | 70366 ( 15.8) | 0.441 | 0.013 |
|  | Betablockers | 43675 ( 54.8) | 77218 ( 17.3) | 0.846 | 0.007 |
|  | Ezetimib | 3107 ( 3.9) | 1859 ( 0.4) | 0.241 | 0.001 |
|  | Other lipid lowering | 407 ( 0.5) | 1565 ( 0.4) | 0.024 | 0.001 |
|  | Insulin | 9619 ( 12.1) | 7961 ( 1.8) | 0.413 | 0.006 |
|  | Metformin | 17043 ( 21.4) | 12655 ( 2.8) | 0.592 | 0.001 |
|  | Sulfonylureas | 1992 ( 2.5) | 1574 ( 0.4) | 0.182 | 0.016 |
|  | Glucosidase inhibitors | 34 ( 0.0) | 49 ( 0.0) | 0.019 | 0.008 |
|  | Pioglitazone | 233 ( 0.3) | 142 ( 0.0) | 0.065 | 0.006 |
|  | DPP4 inhibitors | 5006 ( 6.3) | 3437 ( 0.8) | 0.302 | 0.008 |
|  | GLP1 receptor agonists | 2253 ( 2.8) | 1247 ( 0.3) | 0.207 | 0.001 |
|  | SGLT2 inhibitors | 2885 ( 3.6) | 1303 ( 0.3) | 0.242 | 0.028 |
|  | Neuroleptics | 2207 ( 2.8) | 11654 ( 2.6) | 0.009 | 0.001 |
|  | Anti-depressants | 15459 ( 19.4) | 66880 ( 15.0) | 0.116 | 0.001 |
|  | Anxiolytics | 8359 ( 10.5) | 39130 ( 8.8) | 0.057 | 0.001 |
|  | Sedative | 16864 ( 21.1) | 64162 ( 14.4) | 0.177 | 0.006 |
|  | P2Y12 receptor inhibitors | 9576 ( 12.0) | 4439 ( 1.0) | 0.458 | 0.006 |
|  | ASA cardiac | 34037 ( 42.7) | 32322 ( 7.3) | 0.897 | 0.003 |
|  | NOACs | 12267 ( 15.4) | 28914 ( 6.5) | 0.288 | 0.001 |
|  | Vitamin K antagonists | 6084 ( 7.6) | 11718 ( 2.6) | 0.228 | 0.004 |
|  | Respiratory drugs | 11525 ( 14.4) | 46065 ( 10.3) | 0.125 | 0.004 |

Footnotes - ATT: average treatment effect for the treated, TIA: transient ischemic attack, ACEI: angiotension converting enzyme inhibitors, ARB: angiotension receptor blockers, CCB: calcium channel blockers, NOACs: novel oral anticoagulants, DPP-4: dipeptidyl peptidase 4 inhibitors, SGLT2: sodium-glucose co-transporter-2

**S4 Table.** Demographic and socioeconomic characteristics, comorbidities and prior medication of the *onset population cohort* on their index date, with standardized mean differences (SMD) before and after ATT weighting, by statin use status (presented as n (%))

| **Characteristics** | | **Statin users** | **Nonusers** | **SMD**  **(before ATT weighting)** | **SMD**  **(after ATT weighting)** |
| --- | --- | --- | --- | --- | --- |
| N | | 52128 | 242785 |  |  |
| Women | | 21986 (42.2%) | 135311 (55.7%) | 0.274 | 0.023 |
| Age, years (mean±SD) | | 67.61±13.06 | 55.30±12.84 | 0.951 | 0.012 |
| Age group (years) | | | | | |
|  | 40-49 | 4399 (8.4%) | 96650 (39.8%) | 1.055 | 0.023 |
|  | 50-59 | 11481 (22.0%) | 79891 (32.9%) |  |  |
|  | 60-69 | 12893 (24.7%) | 33831 (13.9%) |  |  |
|  | 70-79 | 12023 (23.1%) | 14035 (5.8%) |  |  |
|  | 80-89 | 9210 (17.7%) | 11685 (4.8%) |  |  |
|  | 90+ | 2122 (4.1%) | 6693 (2.8%) |  |  |
| Education level | | | | | |
|  | Low (Primary school) | 15081 (29.6%) | 34341 (14.3%) | 0.428 | 0.008 |
|  | Medium (Secondary school) | 22545 (44.2%) | 105633 (44.1%) |  |  |
|  | High (Postgraduate) | 13383 (26.2%) | 99692 (41.6%) |  |  |
| Unemployed | | 27526 (52.8%) | 47621 (19.7%) | 0.736 | 0.008 |
| Married | | 27241 (52.3%) | 133421 (55.0%) | 0.054 | 0.009 |
| Disposable income, SEK (Mean±SD) | | 2889.2±10920.9 | 3543.3±11027.8 | 0.060 | 0.007 |
| Country of birth | |  | | |  |
|  | Sweden | 36865 (70.7%) | 182397 (75.1%) | 0.125 | 0.082 |
|  | Nordic | 2284 (4.4%) | 6197 (2.6%) |  |  |
|  | EU | 1625 (3.1%) | 7638 (3.1%) |  |  |
|  | Rest of the world | 11354 (21.8%) | 46553 (19.2%) |  |  |
| **Comorbidities** | | | | | |
|  | Hypertension | 24099 (46.2%) | 23029 (9.5%) | 0.899 | 0.028 |
|  | Cardiovascular disease | 20090 (38.5%) | 19506 (8.0%) | 0.774 | 0.043 |
|  | Ischemic heart disease | 12507 (24.0%) | 3488 (1.4%) | 0.720 | 0.069 |
|  | Stroke or TIA | 6539 (12.5%) | 3123 (1.3%) | 0.455 | 0.008 |
|  | Arrhythmia | 9249 (17.7%) | 11357 (4.7%) | 0.423 | <0.001 |
|  | Heart failure | 6230 (12.0%) | 5339 (2.2%) | 0.387 | 0.026 |
|  | Diabetes | 14410 (27.6%) | 6513 (2.7%) | 0.742 | 0.007 |
|  | Chronic kidney disease | 3504 (6.7%) | 2634 (1.1%) | 0.294 | 0.002 |
|  | Respiratory disease | 3436 (6.6%) | 3749 (1.5%) | 0.258 | 0.012 |
|  | Cancer | 7277 (14.0%) | 16169 (6.7%) | 0.242 | <0.001 |
|  | Liver disease | 258 (0.5%) | 624 (0.3%) | 0.039 | 0.006 |
|  | Obesity | 2714 (5.2%) | 4690 (1.9%) | 0.177 | 0.002 |
|  | Neurological disease | 1210 (2.3%) | 3023 (1.2%) | 0.081 | 0.004 |
|  | Dementia | 2998 (5.8%) | 5590 (2.3%) | 0.176 | 0.018 |
|  | Autoimmune | 3928 (7.5%) | 7749 (3.2%) | 0.194 | 0.001 |
| **Prior medications** | | | | | |
|  | ACEI | 17425 (33.4%) | 18149 (7.5%) | 0.680 | 0.021 |
|  | ARB | 19137 (36.7%) | 24371 (10.0%) | 0.664 | 0.018 |
|  | CCB | 19656 (37.7%) | 25123 (10.3%) | 0.676 | 0.006 |
|  | Diuretics | 18986 (36.4%) | 33923 (14.0%) | 0.535 | 0.013 |
|  | Betablockers | 25465 (48.9%) | 28155 (11.6%) | 0.888 | 0.041 |
|  | Ezetimib | 3348 (6.4%) | 545 (0.2%) | 0.351 | 0.032 |
|  | Other lipid lowering | 775 (1.5%) | 941 (0.4%) | 0.114 | 0.041 |
|  | Insulin | 8300 (15.9%) | 3467 (1.4%) | 0.533 | 0.016 |
|  | Metformin | 15385 (29.5%) | 6879 (2.8%) | 0.777 | 0.005 |
|  | Sulfonylureas | 2049 (3.9%) | 715 (0.3%) | 0.255 | 0.005 |
|  | Glucosidase inhibitors | 34 (0.1%) | 38 (0.0%) | 0.025 | 0.016 |
|  | Pioglitazone | 283 (0.5%) | 78 (0.0%) | 0.096 | 0.007 |
|  | DPP4 inhibitors | 4735 (9.1%) | 1620 (0.7%) | 0.398 | 0.001 |
|  | GLP1 agonists | 3196 (6.1%) | 1201 (0.5%) | 0.319 | <0.001 |
|  | SGLT2 inhibitors | 4131 (7.9%) | 1068 (0.4%) | 0.381 | 0.020 |
|  | Neuroleptics | 2861 (5.5%) | 7505 (3.1%) | 0.119 | 0.035 |
|  | Anti-depressants | 14810 (28.4%) | 45848 (18.9%) | 0.226 | <0.001 |
|  | Anxiolytics | 9986 (19.2%) | 29817 (12.3%) | 0.190 | 0.001 |
|  | Sedative | 14044 (26.9%) | 37817 (15.6%) | 0.280 | 0.003 |
|  | P2Y12 inhibitors | 7517 (14.4%) | 1204 (0.5%) | 0.550 | 0.061 |
|  | ASA cardiac | 20685 (39.7%) | 8888 (3.7%) | 0.972 | 0.052 |
|  | NOACs | 7698 (14.8%) | 10687 (4.4%) | 0.358 | <0.001 |
|  | Vitamin K antagonists | 3239 (6.2%) | 3119 (1.3%) | 0.262 | 0.003 |
|  | Respiratory drugs | 11559 (22.2%) | 37586 (15.5%) | 0.172 | 0.003 |

Footnotes - ATT: average treatment effect for the treated, TIA: transient ischemic attack, ACEI: angiotension converting enzyme inhibitors, ARB: angiotension receptor blockers, CCB: calcium channel blockers, NOACs: novel oral anticoagulants, DPP-4: dipeptidyl peptidase 4 inhibitors, SGLT2: sodium-glucose co-transporter-2

**S5 Table.** Demographic and socioeconomic characteristics, comorbidities and prior medication of the *hospitalised population cohort* on their index date, with standardized mean differences (SMD) before and after ATT weighting, by statin use status (presented as n (%))

| **Characteristics** | | **Statin users** | **Nonusers** | **SMD**  **(before ATT weighting)** | **SMD**  **(after ATT weighting)** |
| --- | --- | --- | --- | --- | --- |
| N | | 15246 | 24846 |  |  |
| Women | | 5731 (37.6%) | 11352 (45.7%) | 0.165 | 0.009 |
| Age, years (mean±SD) | | 73.5±11.4 | 67.3±15.3 | 0.458 | 0.053 |
| Age group (years) | |  |  |  |  |
|  | 40–49 | 474 (3.1%) | 3683 (14.8%) | 0.629 | 0.039 |
|  | 50-59 | 1532 (10.0%) | 5254 (21.1%) |  |  |
|  | 60-69 | 3014 (19.8%) | 4597 (18.5%) |  |  |
|  | 70-79 | 5029 (33.0%) | 4713 (19.0%) |  |  |
|  | 80-89 | 4350 (28.5%) | 4609 (18.6%) |  |  |
|  | 90+ | 847 (5.6%) | 1990 (8.0%) |  |  |
| Education level | |  | | |  |
|  | Low (Primary school) | 5640 (38.3%) | 7140 (29.7%) | 0.214 | 0.004 |
|  | Medium (Secondary school) | 6069 (41.2%) | 10151 (42.2%) |  |  |
|  | High (Postgraduate) | 3029 (20.6%) | 6759 (28.1%) |  |  |
| Unemployed | | 11368 (74.6%) | 13780 (55.6%) | 0.407 | 0.012 |
| Married | | 7093 (46.5%) | 11431 (46.0%) | 0.010 | 0.010 |
| Disposable income, SEK (mean±SD) | | 2350.7±5381.6 | 2653.6±4300 | 0.062 | 0.007 |
| Country of birth | |  | | |  |
|  | Sweden | 10453 (68.6%) | 16710 (67.3%) | 0.075 | 0.070 |
|  | Nordic | 844 (5.5%) | 1122 (4.5%) |  |  |
|  | EU | 573 (3.8%) | 857 (3.4%) |  |  |
|  | Rest of the world | 3376 (22.1%) | 6157 (24.8%) |  |  |
| **Comorbidities** | | | | | |
|  | Hypertension | 9493 (62.3%) | 7301 (29.4%) | 0.699 | 0.022 |
|  | Cardiovascular disease | 8128 (53.3%) | 6077 (24.5%) | 0.620 | 0.040 |
|  | Ischemic heart disease | 5125 (33.6%) | 1387 (5.6%) | 0.755 | 0.065 |
|  | Stroke or TIA | 2487 (16.3%) | 1003 (4.0%) | 0.415 | 0.012 |
|  | Arrhythmia | 4225 (27.7%) | 3819 (15.4%) | 0.304 | 0.002 |
|  | Heart failure | 3337 (21.9%) | 2472 (9.9%) | 0.331 | 0.036 |
|  | Diabetes | 5898 (38.7%) | 2405 (9.7%) | 0.720 | 0.021 |
|  | Chronic kidney disease | 2093 (13.7%) | 1298 (5.2%) | 0.293 | 0.008 |
|  | Respiratory disease | 1933 (12.7%) | 1862 (7.5%) | 0.173 | 0.013 |
|  | Cancer | 3098 (20.3%) | 4245 (17.1%) | 0.083 | 0.014 |
|  | Liver disease | 122 (0.8%) | 255 (1.0%) | 0.024 | 0.002 |
|  | Obesity | 1081 (7.1%) | 886 (3.6%) | 0.157 | 0.009 |
|  | Neurological disease | 509 (3.3%) | 918 (3.7%) | 0.019 | 0.007 |
|  | Dementia | 917 (6.0%) | 1301 (5.2%) | 0.034 | 0.010 |
|  | Autoimmune | 1866 (12.2%) | 2017 (8.1%) | 0.137 | <0.001 |
| **Prior medications** | | | | | |
|  | ACEI | 5702 (37.4%) | 3955 (15.9%) | 0.501 | 0.006 |
|  | ARB | 6230 (40.9%) | 5009 (20.2%) | 0.461 | 0.032 |
|  | CCB | 6814 (44.7%) | 5598 (22.5%) | 0.483 | 0.010 |
|  | Diuretics | 7731 (50.7%) | 7799 (31.4%) | 0.401 | 0.004 |
|  | Betablockers | 9507 (62.4%) | 6823 (27.5%) | 0.749 | 0.023 |
|  | Ezetimib | 886 (5.8%) | 169 (0.7%) | 0.293 | 0.001 |
|  | Other lipid lowering | 204 (1.3%) | 210 (0.8%) | 0.047 | 0.013 |
|  | Insulin | 3434 (22.5%) | 1229 (4.9%) | 0.528 | 0.003 |
|  | Metformin | 4887 (32.1%) | 1916 (7.7%) | 0.640 | 0.001 |
|  | Sulfonylureas | 810 (5.3%) | 283 (1.1%) | 0.238 | 0.024 |
|  | Glucosidase inhibitors | 13 (0.1%) | 6 (0.0%) | 0.026 | 0.014 |
|  | Pioglitazone | 87 (0.6%) | 21 (0.1%) | 0.085 | 0.058 |
|  | DPP4 inhibitors | 1814 (11.9%) | 584 (2.4%) | 0.378 | 0.018 |
|  | GLP1 agonists | 1040 (6.8%) | 294 (1.2%) | 0.291 | 0.023 |
|  | SGLT2 inhibitors | 1284 (8.4%) | 311 (1.3%) | 0.339 | 0.033 |
|  | Neuroleptics | 895 (5.9%) | 1624 (6.5%) | 0.028 | 0.018 |
|  | Anti-depressants | 4829 (31.7%) | 6490 (26.1%) | 0.123 | 0.002 |
|  | Anxiolytics | 3308 (21.7%) | 4832 (19.4%) | 0.056 | 0.001 |
|  | Sedative | 4959 (32.5%) | 6651 (26.8%) | 0.126 | 0.012 |
|  | P2Y12 inhibitors | 2798 (18.4%) | 442 (1.8%) | 0.573 | 0.053 |
|  | ASA cardiac | 7348 (48.2%) | 2799 (11.3%) | 0.883 | 0.020 |
|  | NOACs | 3303 (21.7%) | 3324 (13.4%) | 0.219 | 0.003 |
|  | Vitamin K antagonists | 1530 (10.0%) | 1197 (4.8%) | 0.200 | 0.001 |
|  | Respiratory drugs | 4294 (28.2%) | 6063 (24.4%) | 0.086 | 0.004 |

Footnotes - ATT: average treatment effect for the treated, TIA: transient ischemic attack, ACEI: angiotension converting enzyme inhibitors, ARB: angiotension receptor blockers, CCB: calcium channel blockers, NOACs: novel oral anticoagulants, DPP-4: dipeptidyl peptidase 4 inhibitors, SGLT2: sodium-glucose co-transporter-2

**S6 Table.** Crude incidence rates per 1000 person-years (with 95% exact Poisson Confidence Intervals (CI)) for various COVID-19 outcomes in the three studied cohorts (overall population cohort, COVID-19 onset cohort and hospitalized cohort), by regular statin users vs non-users

| **Cohort** | **COVID-19 outcomes** | **Regular statin users** | | | **Non-users** | | |
| --- | --- | --- | --- | --- | --- | --- | --- |
|  |  | **Events** | **Person-years** | **Incidence rates**  **[95%CI)** | **Events** | **Person-years** | **Incidence rates (95%CI)** |
|  |  | **N= 79 769** | | | **N= 445 153** | | |
| ***Overall cohort*** | Test-positive | 3812 | 80427 | 47.4 [45.9-48.9] | 25376 | 450753 | 56.3 [55.6-57.0] |
|  | Diagnosis | 4188 | 80289 | 52.2 [50.6-53.8] | 26633 | 450297 | 59.1 [58.4-59.9] |
|  | Hospitalization | 1837 | 80740 | 22.8 [21.7-23.8] | 5717 | 454160 | 12.6 [12.3-12.9] |
|  | ICU admission | 97 | 81184 | 1.20 [1.00-1.50] | 264 | 455684 | 0.60 [0.50-0.70] |
|  | Death | 768 | 81213 | 9.50 [8.80-10.1] | 2722 | 455782 | 6.00 [5.70-6.20] |
| ***Onset cohort*** |  | **N= 29 204** | | | **N= 242 785** | | |
|  | Hospitalization | 9131 | 4748 | 1923.2 [1884-1963.1] | 24857 | 46129 | 538.9 [532.2-545.6] |
|  | ICU admission | 866 | 7855 | 110.3 [103-117.8] | 2701 | 54901 | 49.2 [47.4-51.1] |
|  | Death | 2666 | 7126 | 374.1 [360-388.6] | 6282 | 53522 | 117.4 [114.5-120.3] |
| ***Hospitali-zed cohort*** |  | **N= 9 067** | | | **N= 24 846** | | |
|  | ICU admission | 842 | 3139 | 268.2 [250.4-287] | 2673 | 8910 | 300 [288.7-311.6] |
|  | Death | 1865 | 2763 | 675 [644.7-706.4] | 3386 | 8788 | 385.3 [372.4-398.5] |

**S7 Table.** Hazard ratios (HRs), unadjusted and adjusted using ATT weighting, with 95% Confidence Interval (CI), comparing the risk of various COVID-19 outcomes between *regular statin users and non-users*, in the 3 studied cohorts (overall population cohort, COVID-19 onset cohort and hospitalized cohort).

| ***Overall cohort*** | **Unadjusted HR (95%CI)** | | | **ATT weight-adjusted HR (95%CI)** | | |
| --- | --- | --- | --- | --- | --- | --- |
|  | **Total** | **Men** | **Women** | **Total** | **Men** | **Women** |
| Test-positive | 0.84  [0.81-0.87] | 0.93  [0.89-0.97] | 0.77  [0.73-0.81] | 0.92  [0.87-0.98] | 0.94  [0.87-1.01] | 0.90  [0.83-0.98] |
| Diagnosis | 0.88  [0.86-0.91] | 0.97  0.92-1.01] | 0.81  [0.77-0.85] | 0.92  [0.87-0.97] | 0.93  [0.86-1.00] | 0.91  [0.84-0.99] |
| Hospitalization | 1.81  [1.71-1.9] | 1.82  [1.70-1.94] | 1.67  [1.53-1.82] | 0.88  [0.81-0.96] | 0.88  [0.79-0.98] | 0.87  [0.75-1.02] |
| ICU admission | 2.09  [1.66-2.64] | 1.81  [1.38-2.38] | 2.15  [1.38-3.36] | 0.98  [0.65-1.47] | 0.95  [0.58-1.57] | 1.05  [0.57-1.93] |
| Death | 1.56  [1.44-1.69] | 1.65  [1.49-1.82] | 1.34  [1.17-1.53] | 0.74  [0.66-0.83] | 0.71  [0.62-0.82] | 0.82  [0.69-0.97] |
| ***Onset cohort*** | **Unadjusted HR (95%CI)** | | | **ATT weight-adjusted HR (95%CI)** | | |
|  | **Total** | **Men** | **Women** | **Total** | **Men** | **Women** |
| Hospitalization | 3.47  [3.39-3.56] | 3.03  [2.94-3.13] | 3.78  [3.64-3.93] | 0.92  [0.88-0.96] | 0.88  [0.83-0.93] | 0.99  [0.93-1.05] |
| ICU admission | 2.69  [2.49-2.90] | 2.23  [2.04-2.43] | 2.78  [2.38-3.24] | 0.84  [0.73-0.97] | 0.85  [0.72-1.01] | 0.79  [0.62-1.00] |
| Death | 3.64  [3.48-3.81] | 3.72  [3.51-3.95] | 3.29  [3.06-3.54] | 0.80  [0.75-0.86] | 0.82  [0.74-0.91] | 0.77  [0.69-0.85] |
| ***Hospitalized cohort*** | **Unadjusted HR (95%CI)** | | | **ATT weight-adjusted HR (95%CI)** | | |
|  | **Total** | **Men** | **Women** | **Total** | **Men** | Women |
| ICU admission | 0.86  [0.79-0.92] | 0.81  [0.74-0.88] | 0.81  [0.70-0.95] | 0.99  [0.86-1.13] | 1.03  [0.87-1.21] | 0.87  [0.68-1.11] |
| Death | 1.59  [1.50-1.68] | 1.69  [1.57-1.81] | 1.41  [1.28-1.55] | 0.87  [0.79-0.96] | 0.9  [0.79-1.02] | 0.82  [0.71-0.95] |

Footnote: ATT - Average treatment effect for the treated

**S1 Figure.** Propensity score distributions in the three study cohorts (overall population cohort, COVID-19 onset cohort, hospitalized cohort) before weighting


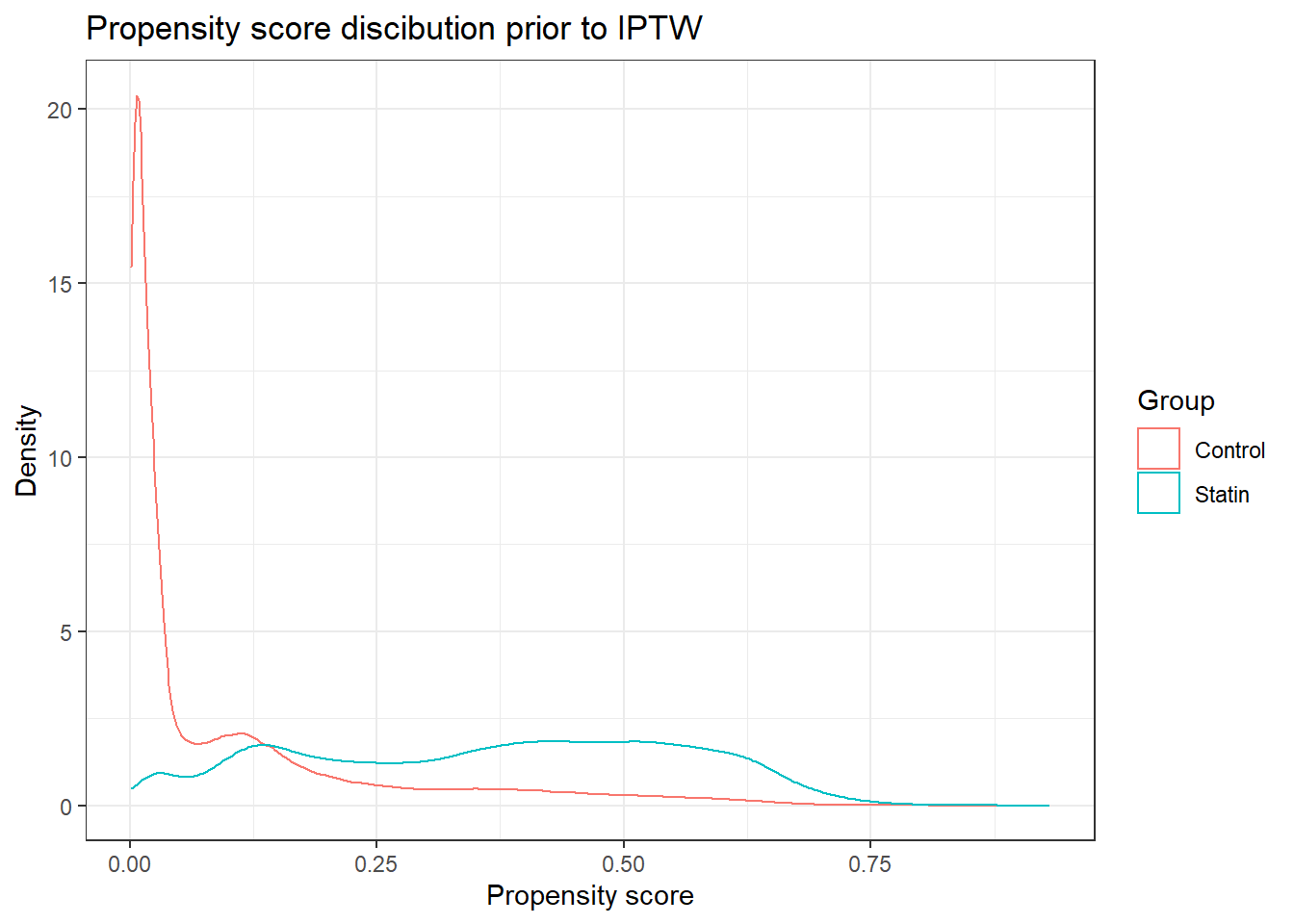


Overall cohort


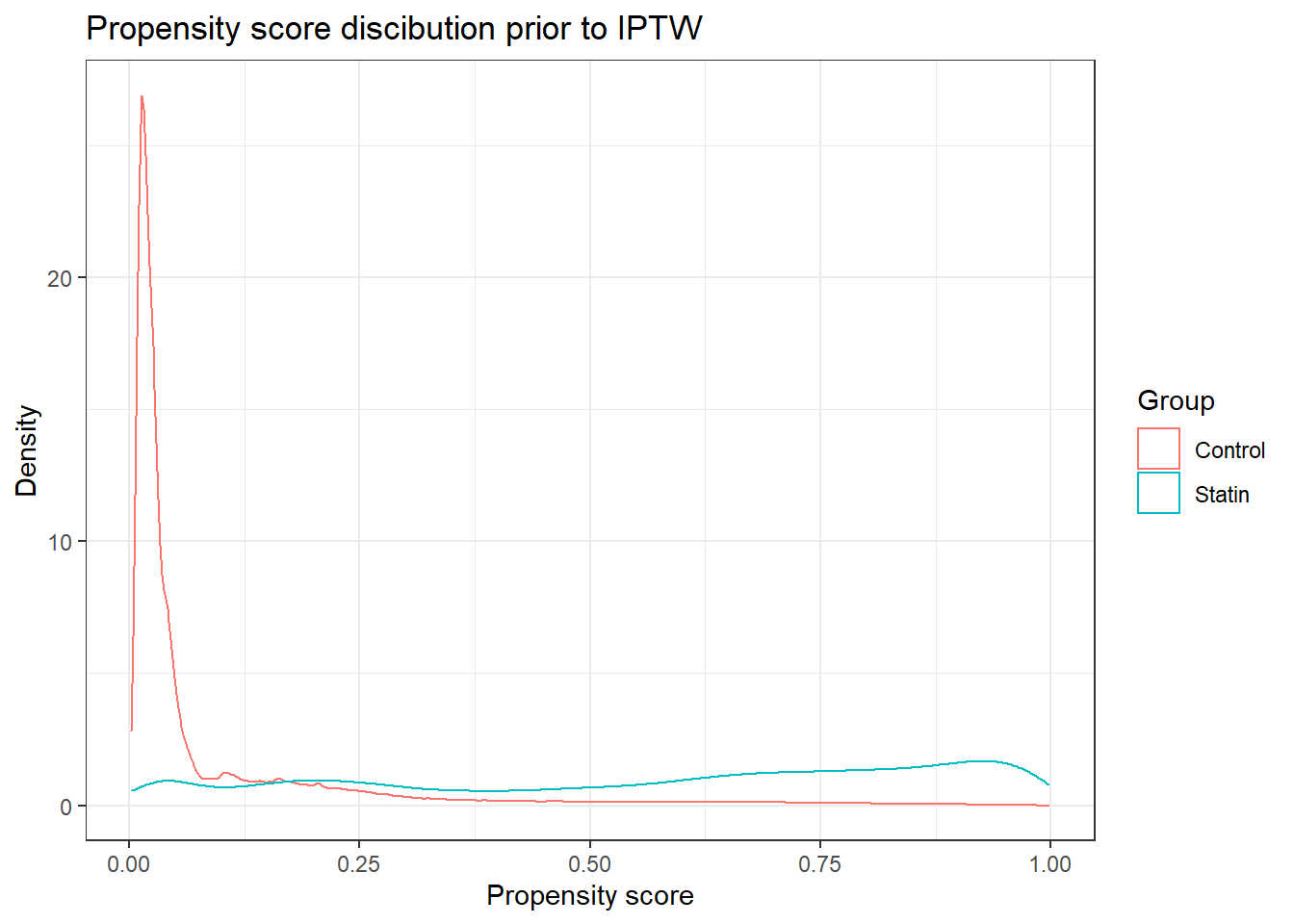


Onset cohort


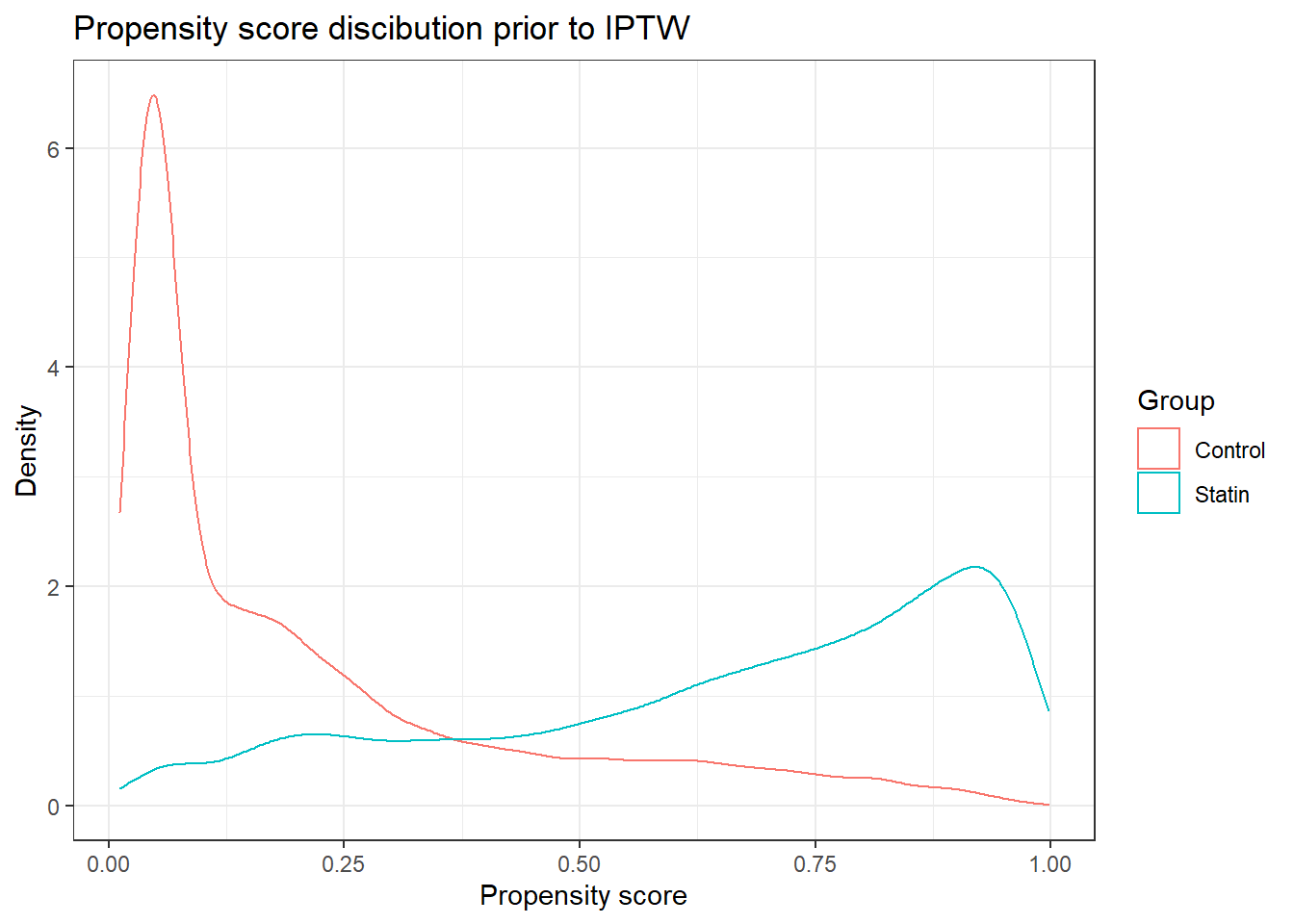


Hospitalized cohort

**S2 Figure.** Cumulative incidence Kaplan Meiers curves for COVID-19 outcomes in prior statin users and nonusers in the *overall population cohort*


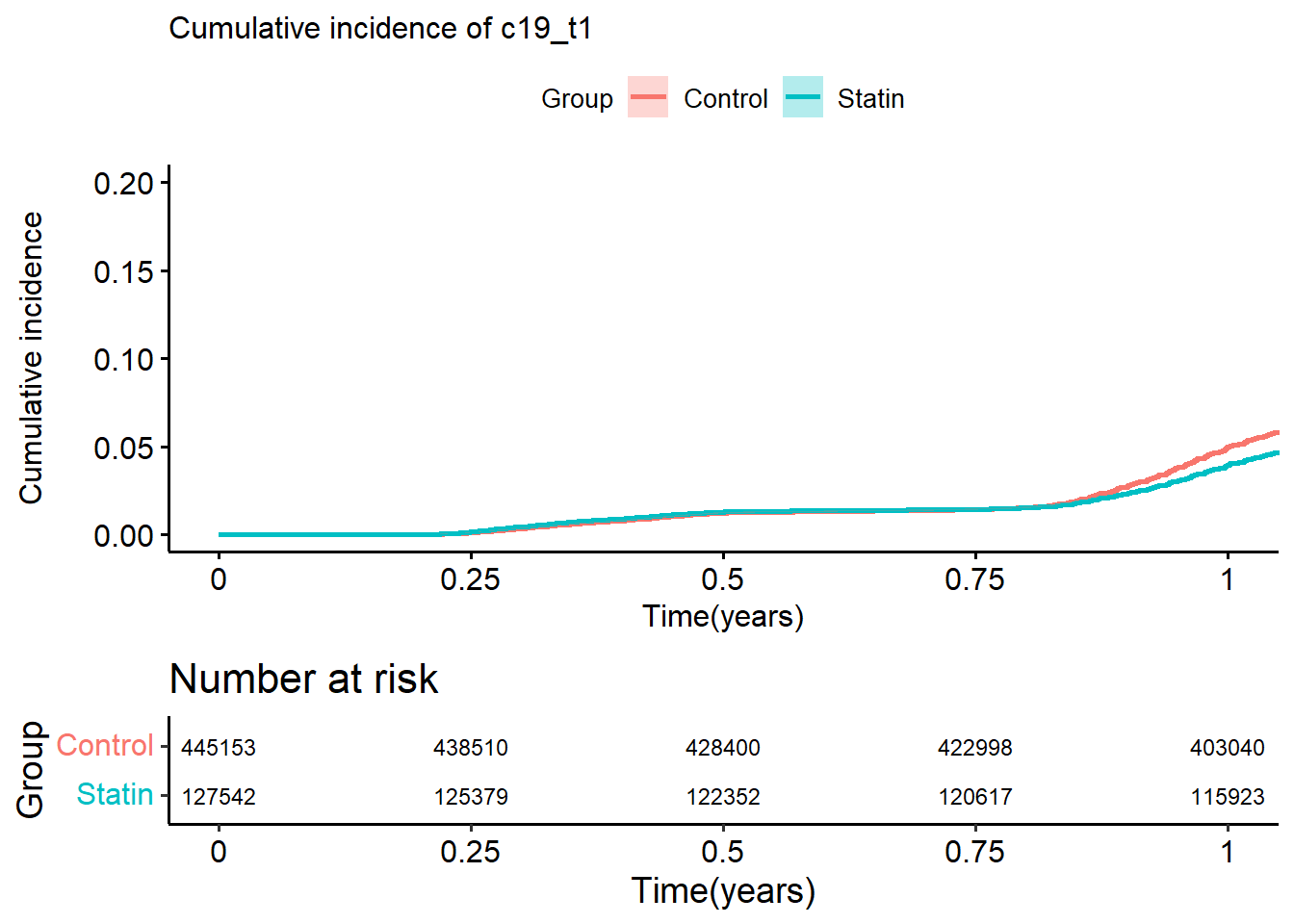

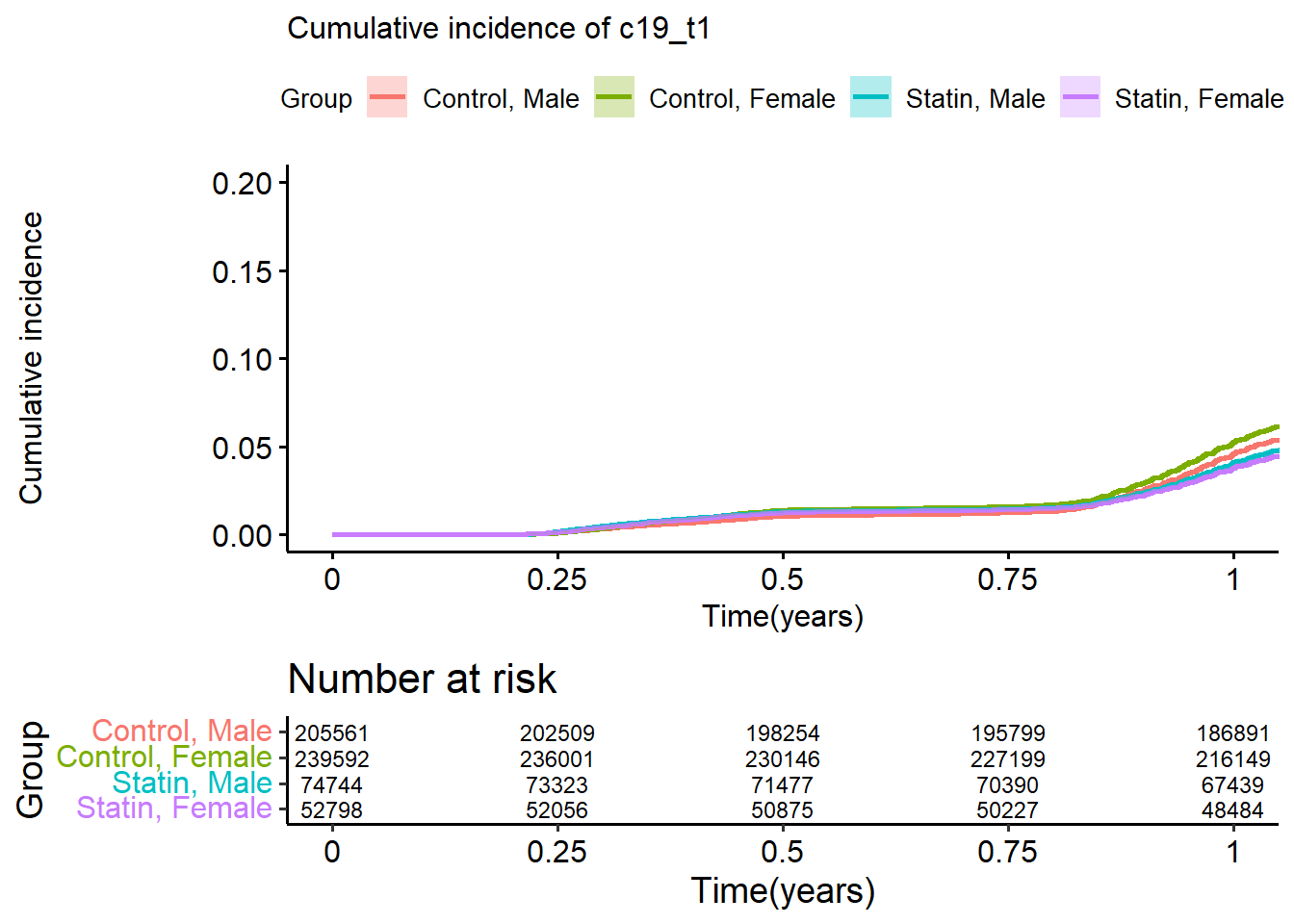


Cumulative incidence of COVID-19 test positivity


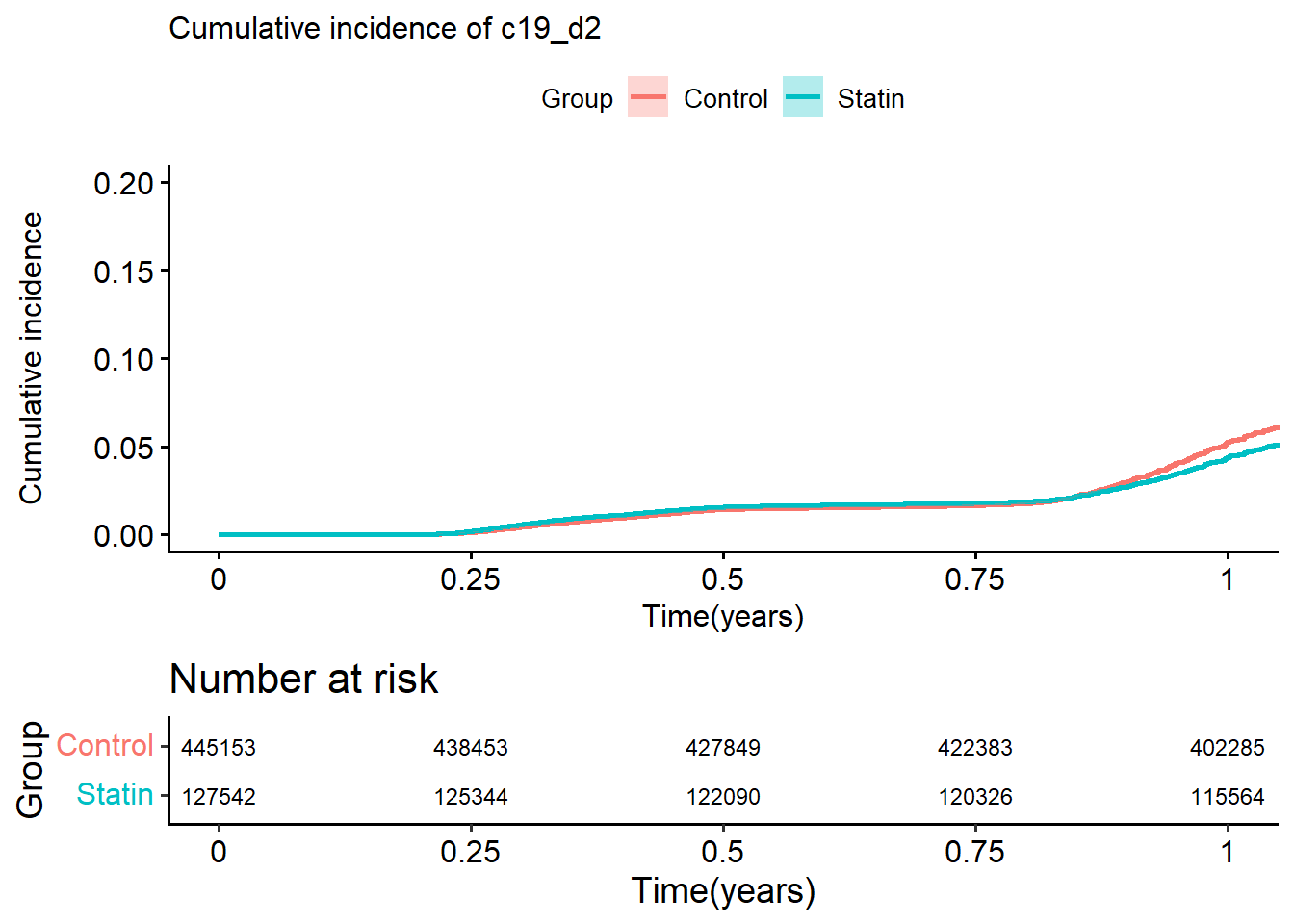

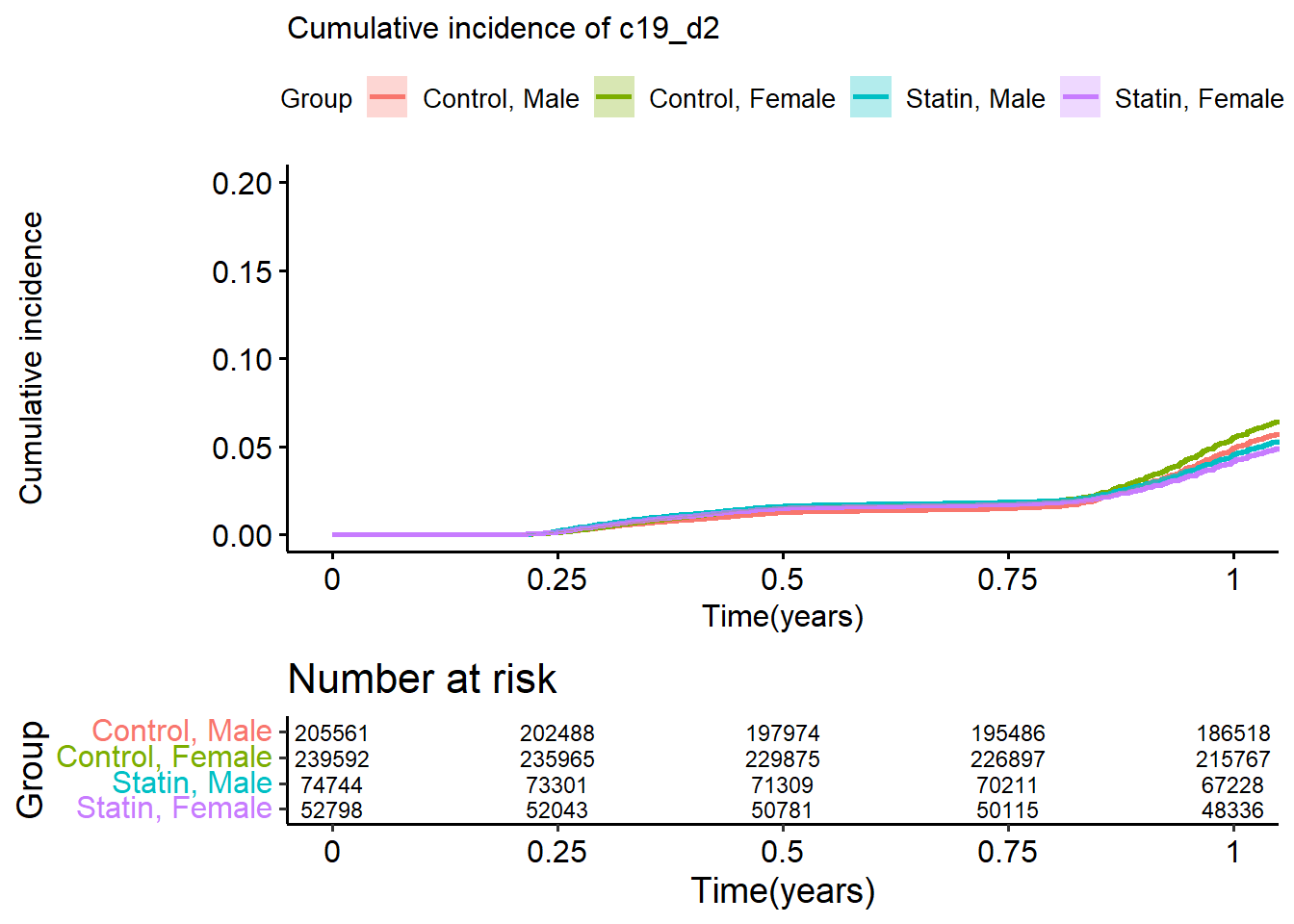


Cumulative incidence of COVID-19 diagnosis


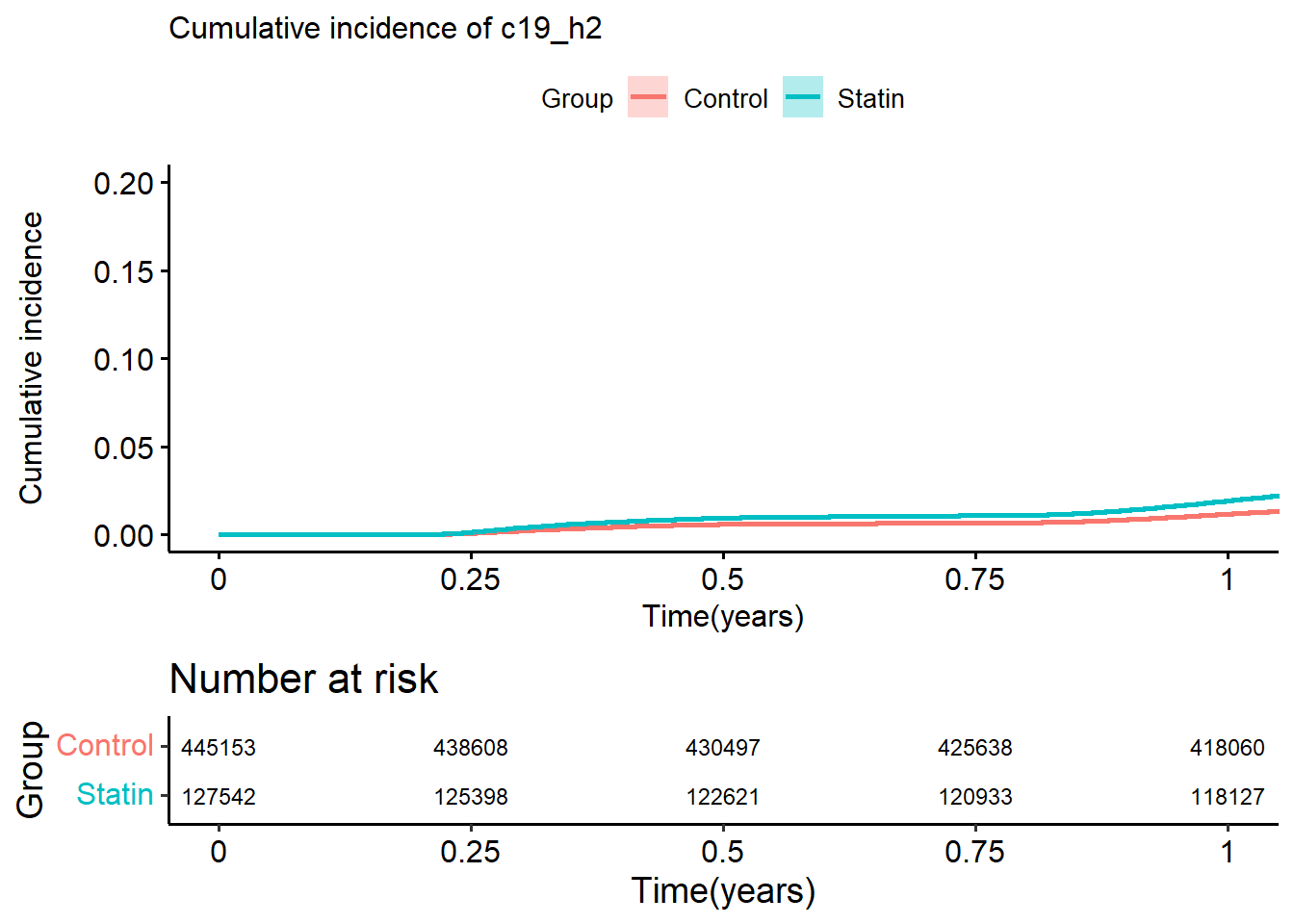

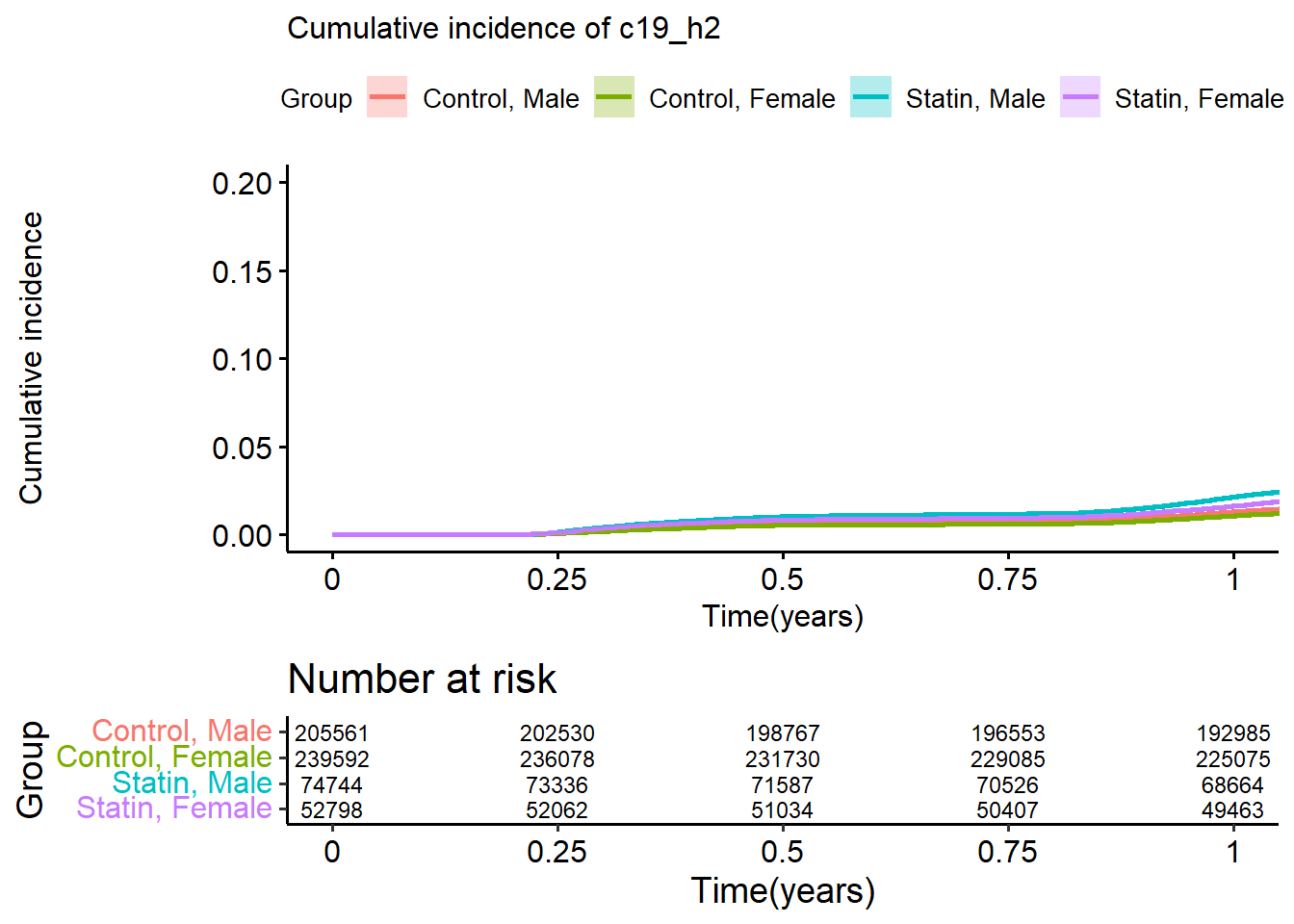


Cumulative incidence of COVID-19 hospitalization


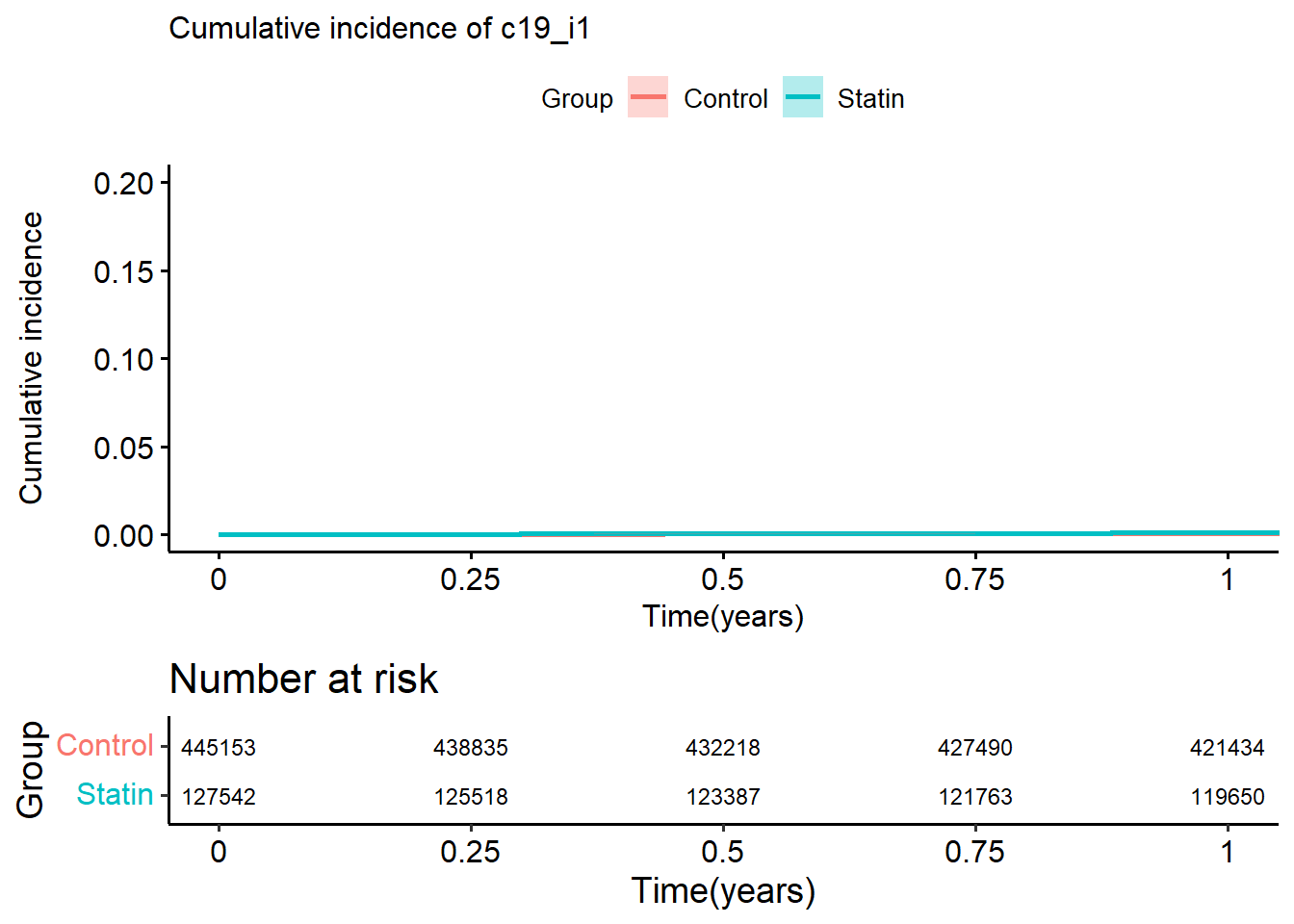

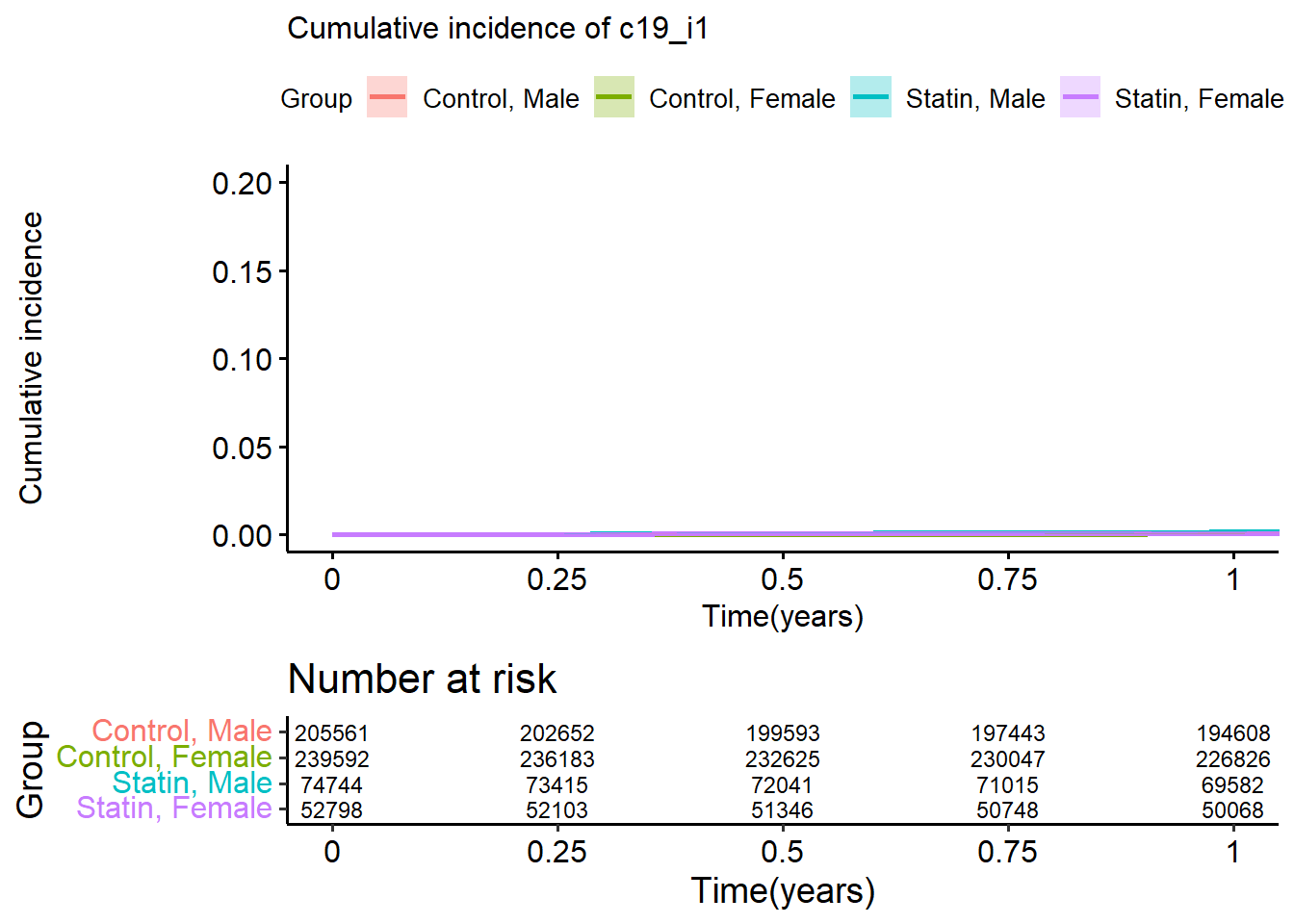


Cumulative incidence of COVID-19 ICU admission


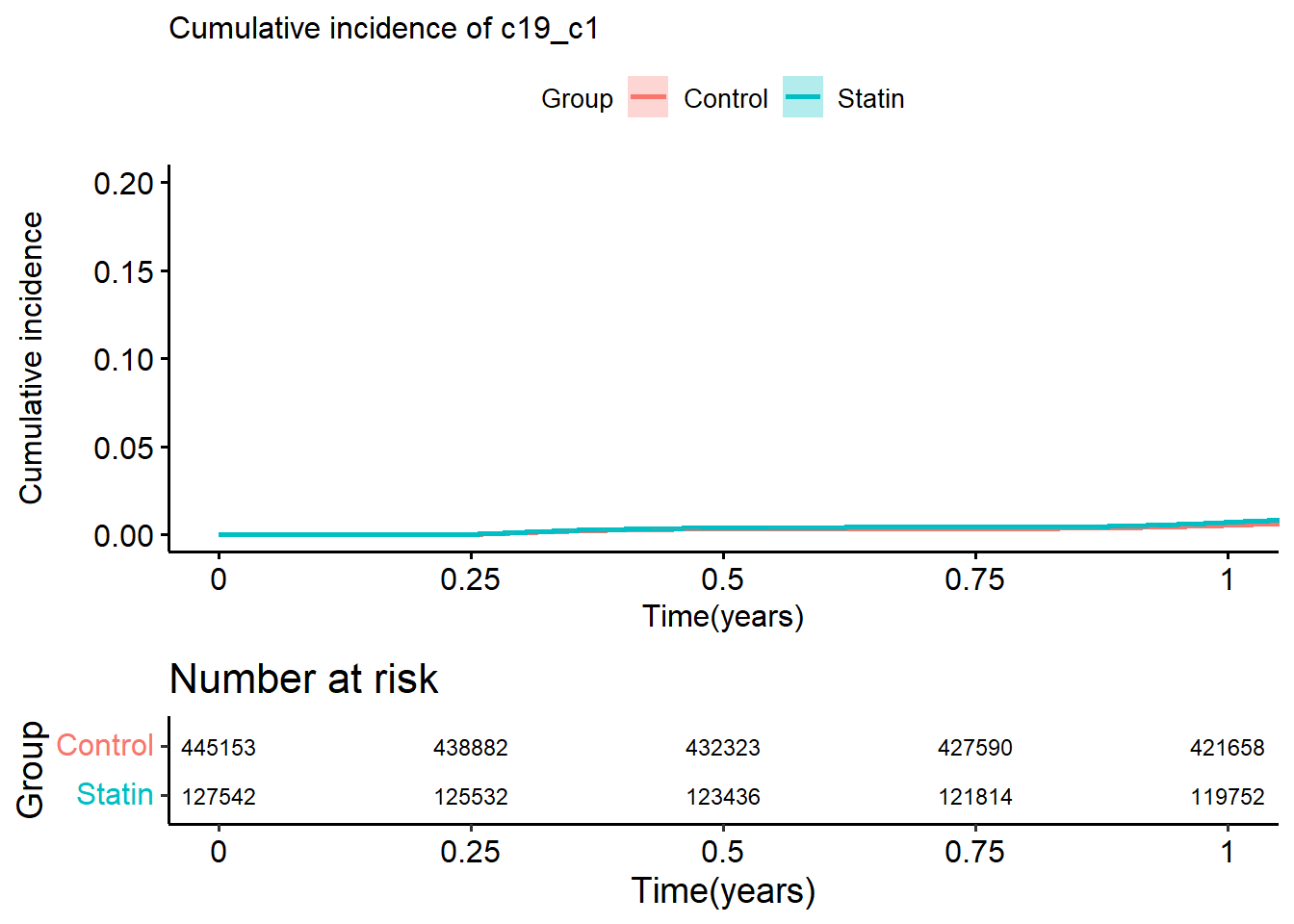

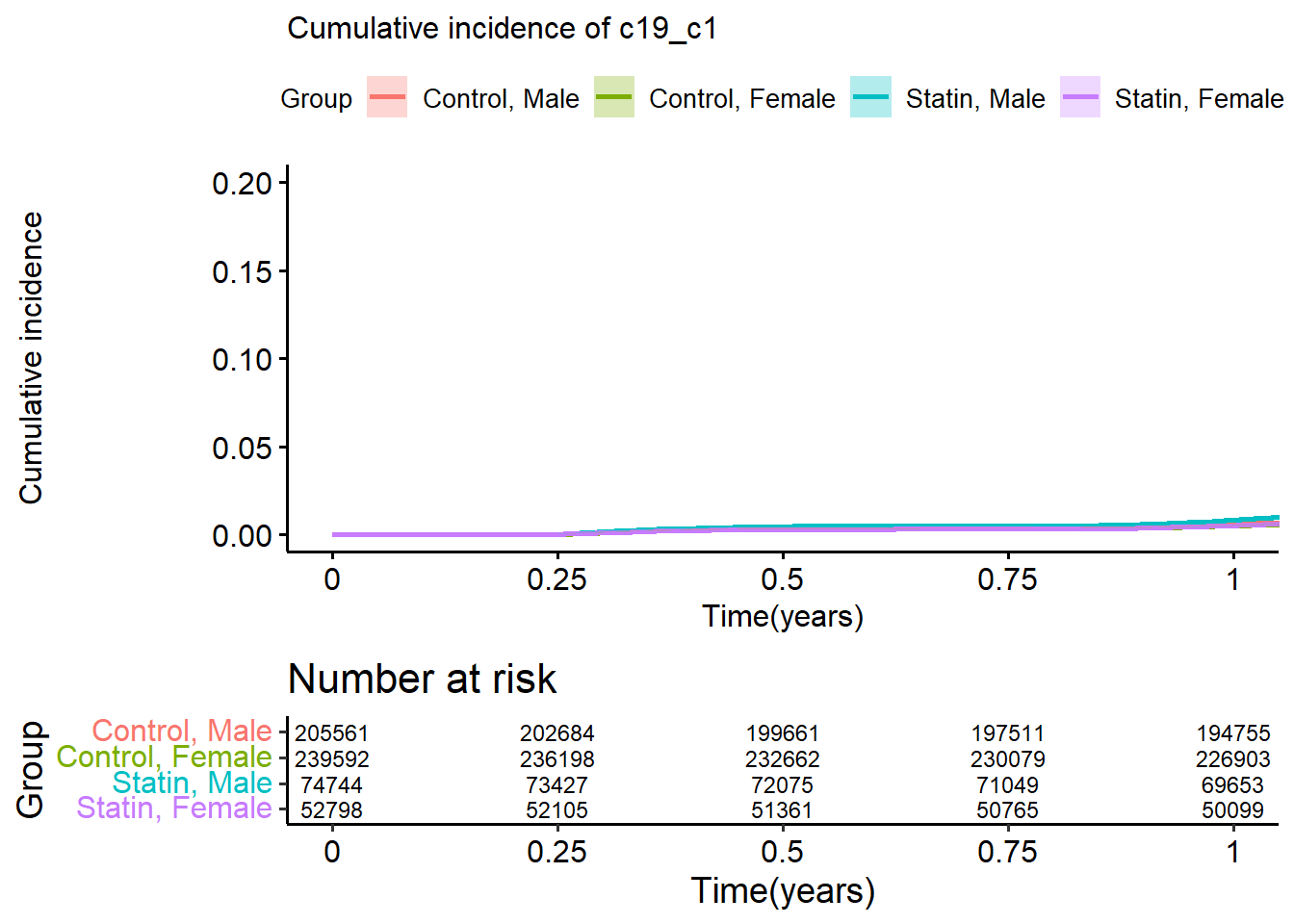


Cumulative incidence of COVID-19 death

**S3 Figure.** Cumulative incidence Kaplan Meiers curves for COVID-19 outcomes in prior statin users and nonusers in the *COVID-19 onset cohort*


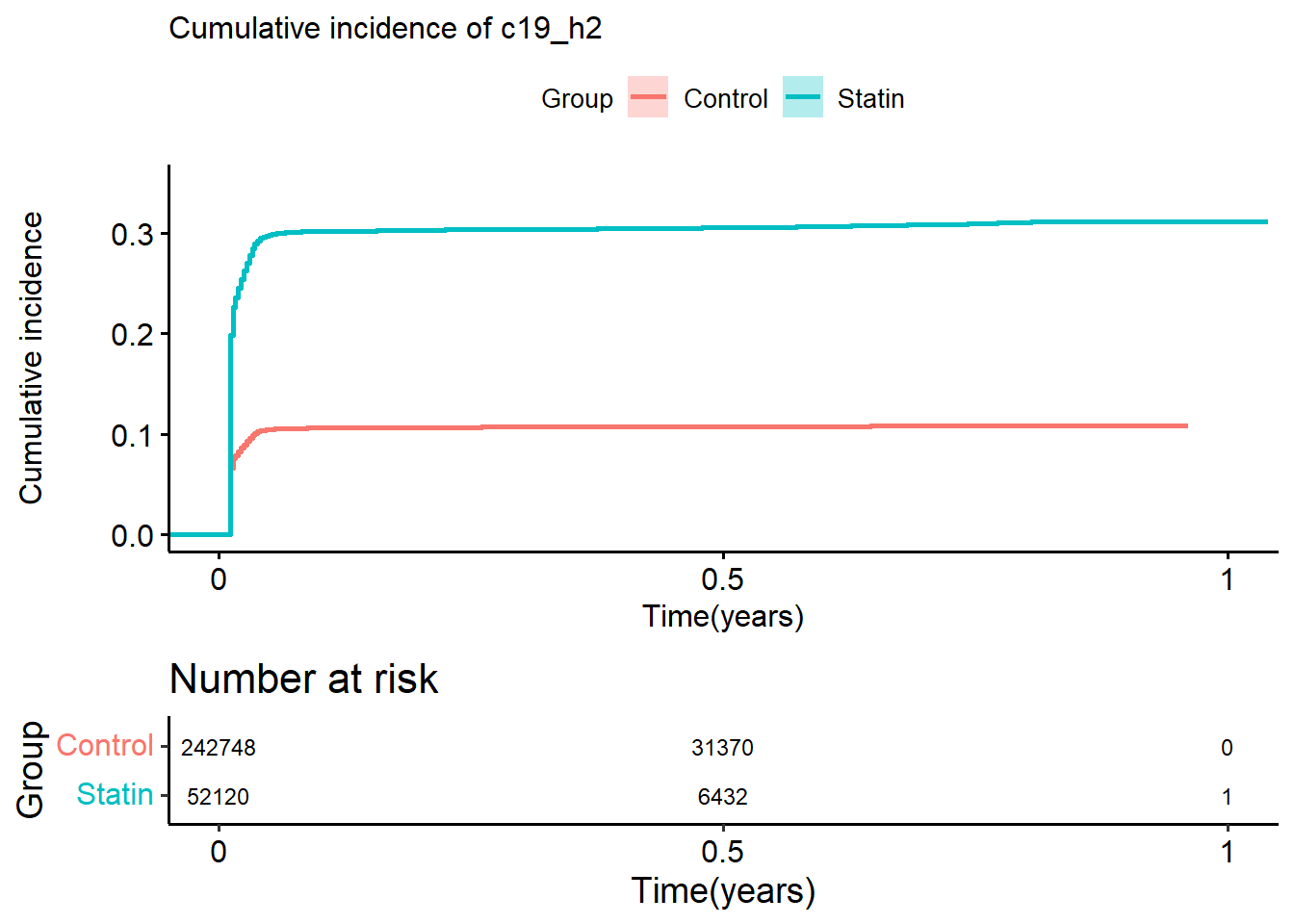

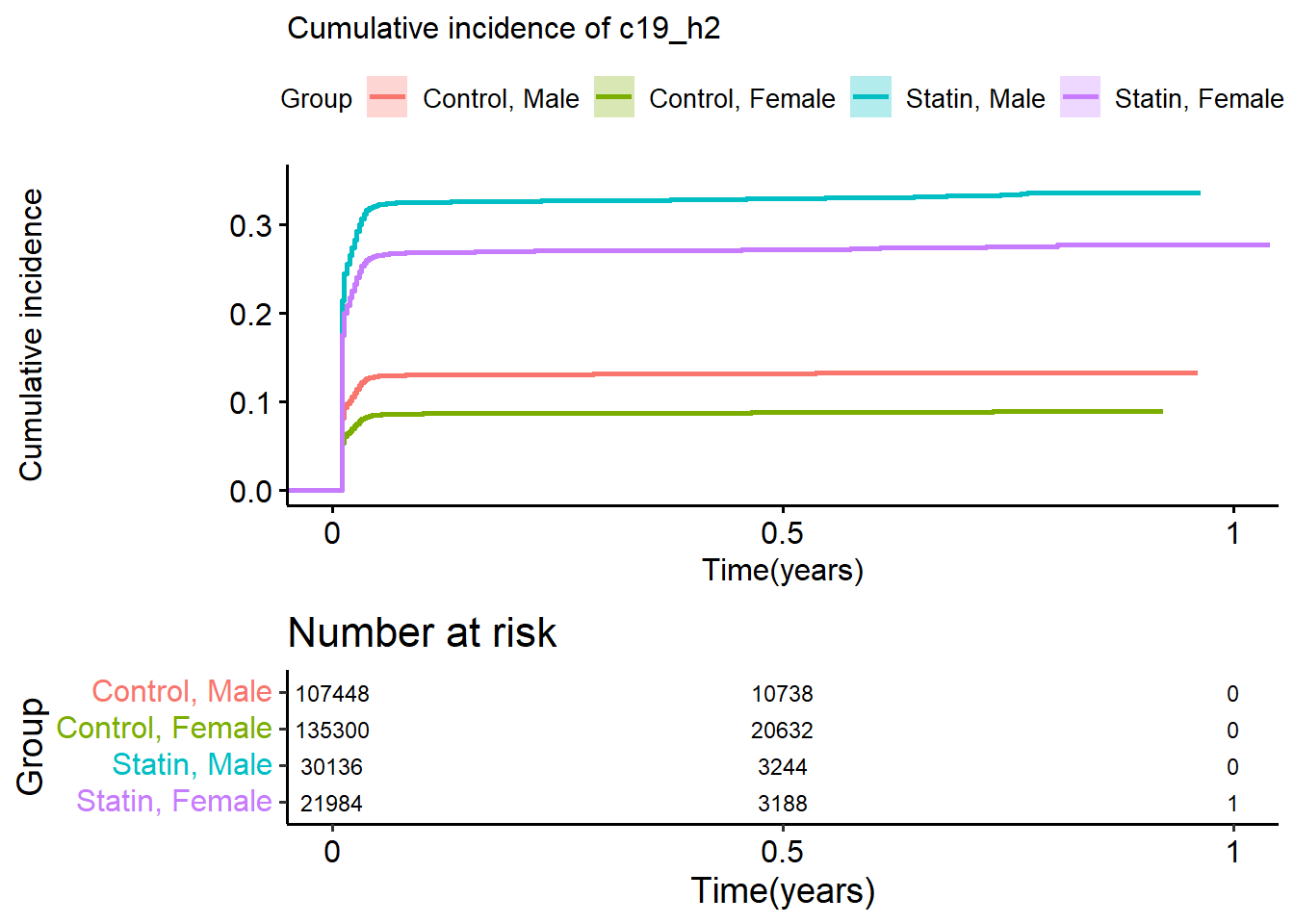


Cumulative incidence of COVID-19 hospitalization


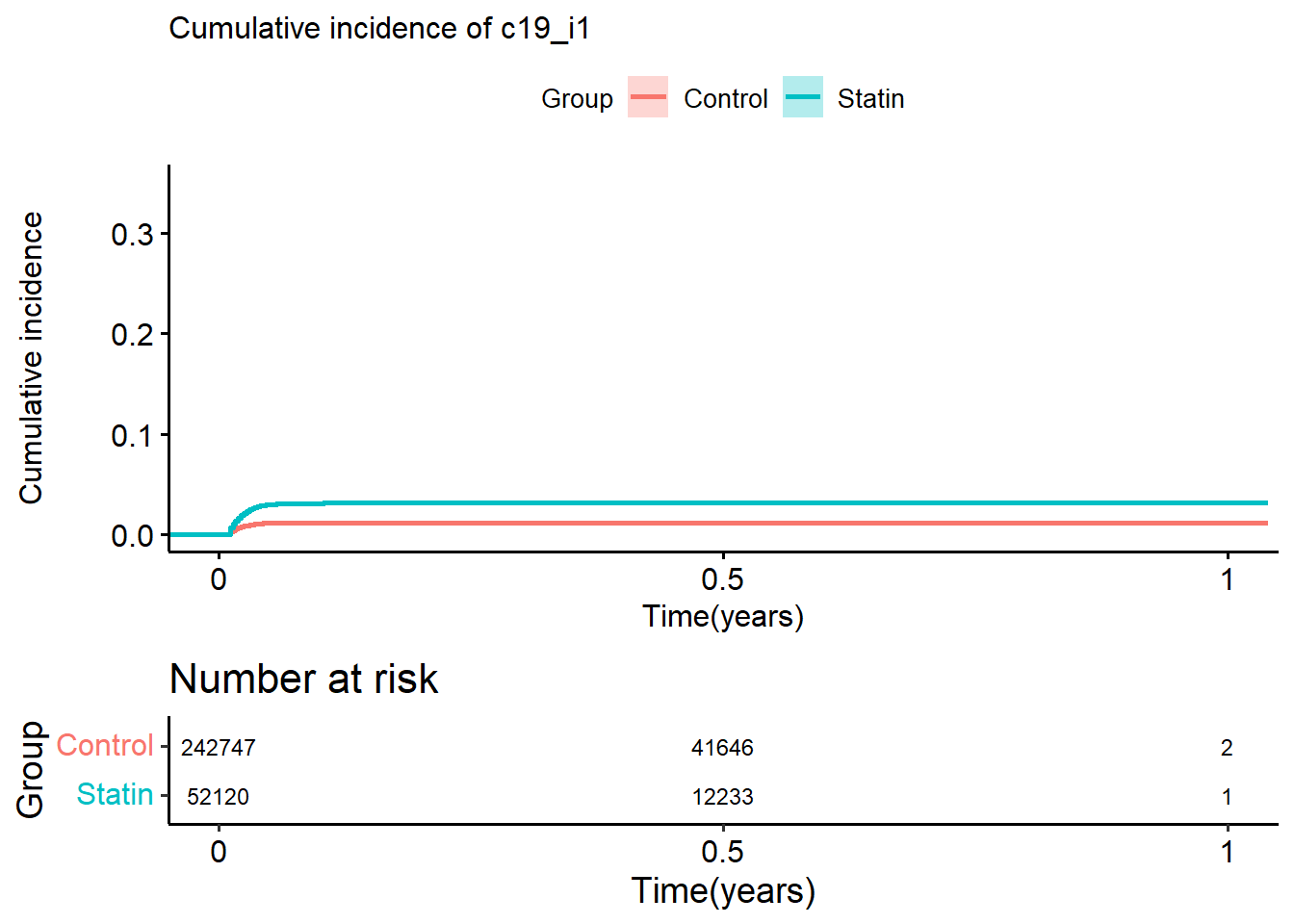

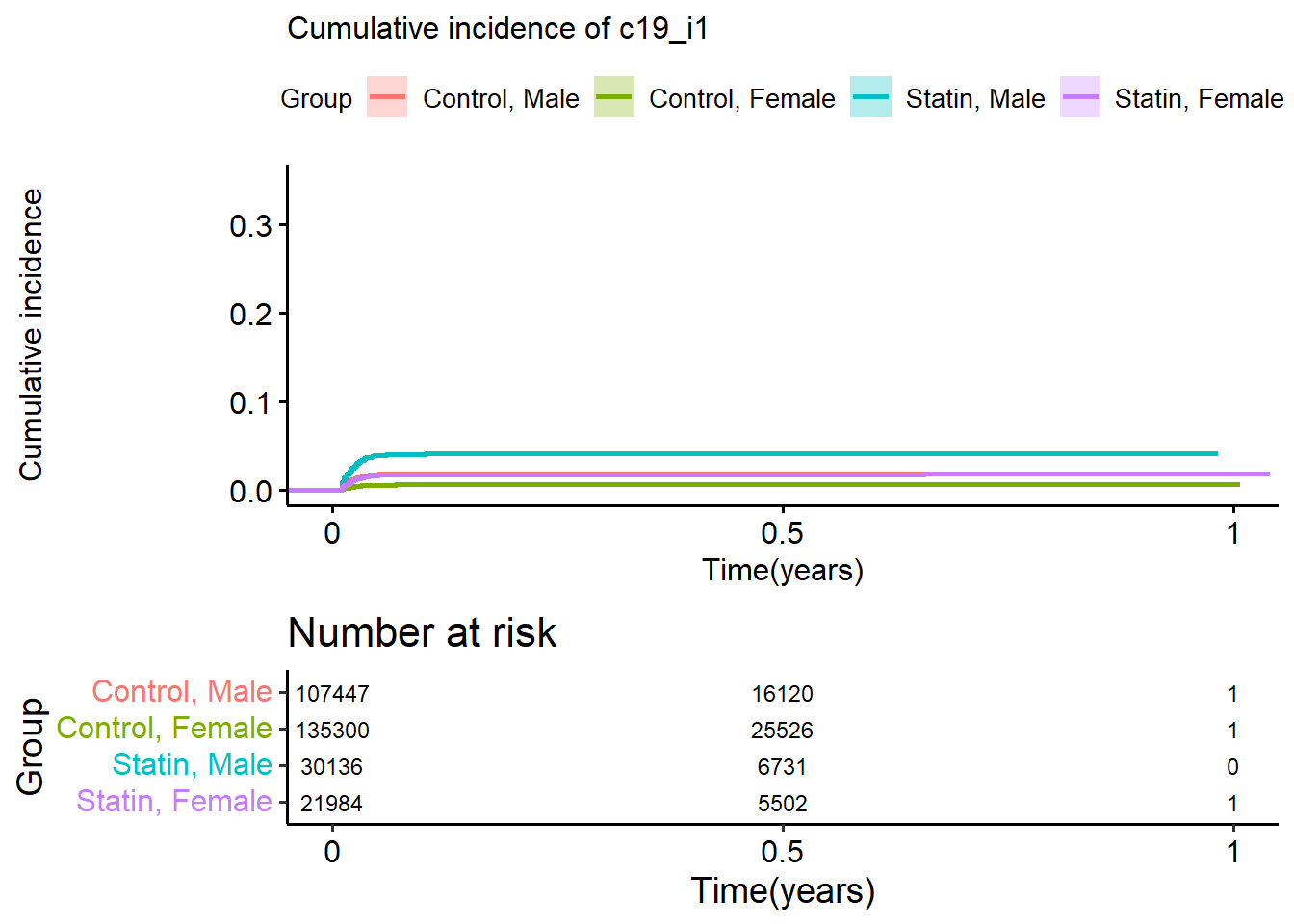


Cumulative incidence of COVID-19 ICU admission


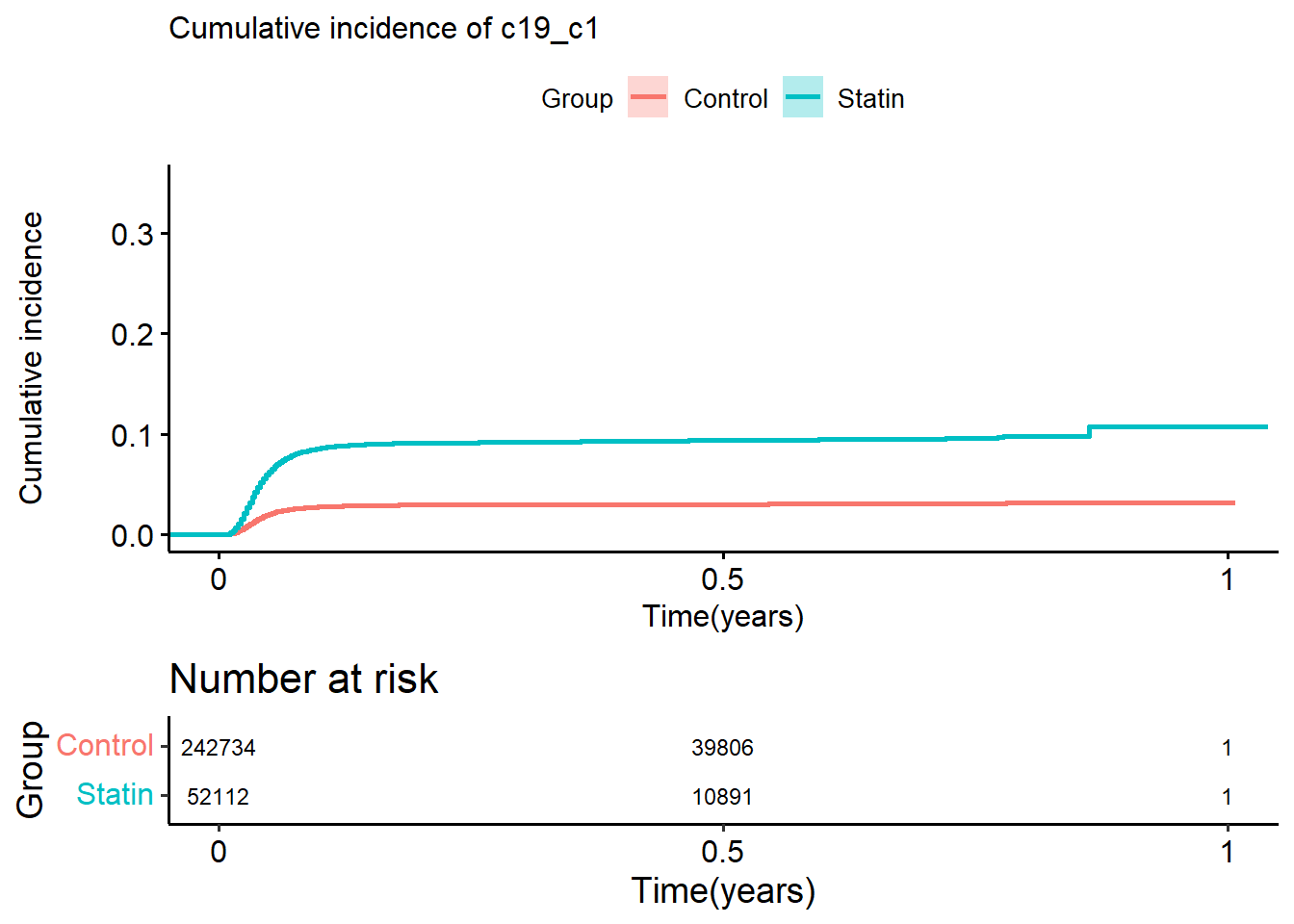

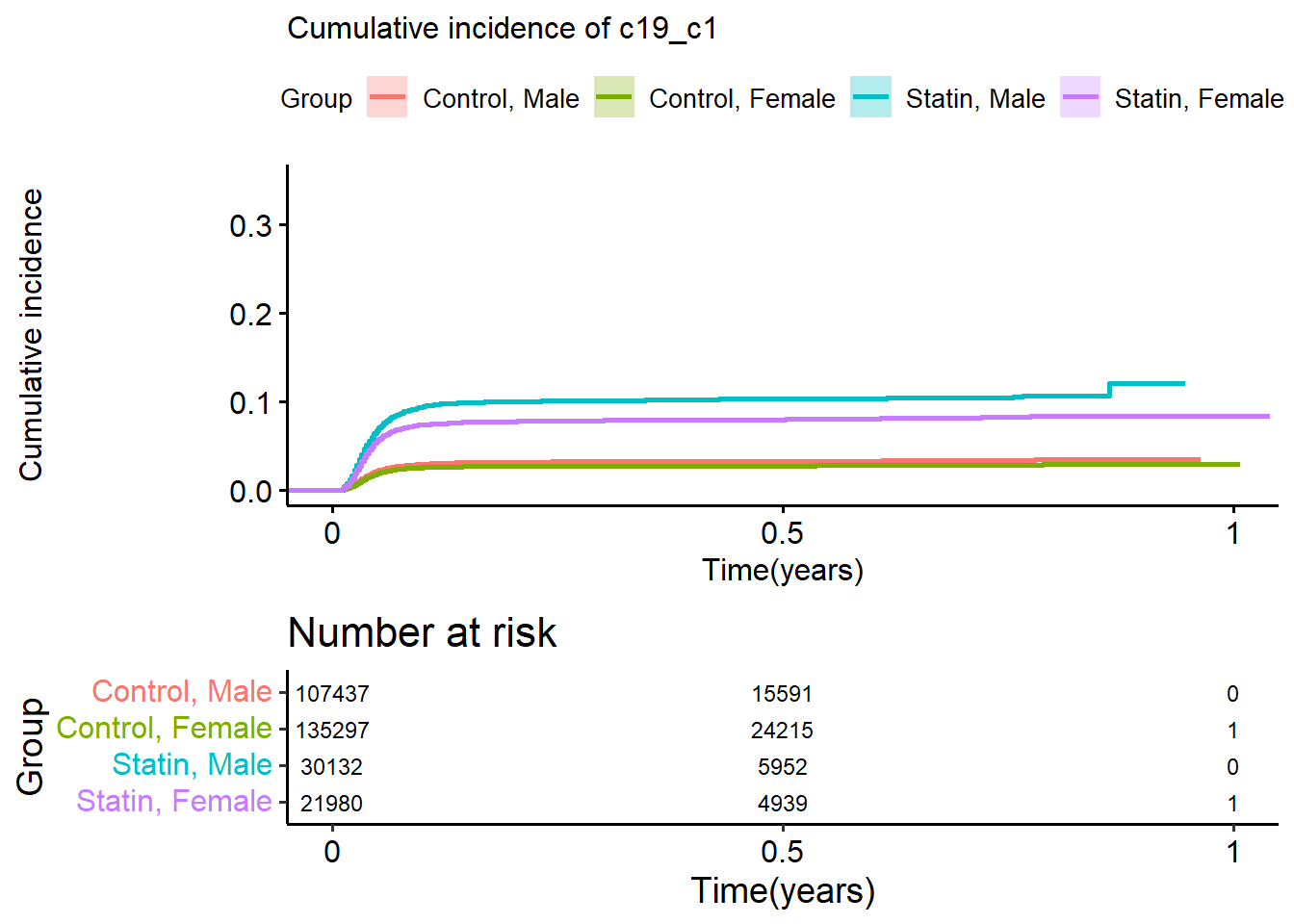


Cumulative incidence of COVID-19 death

**S4 Figure.** Cumulative incidence Kaplan Meiers curves for COVID-19 outcomes in prior statin users and nonusers in the *COVID-19 hospitalized cohort*


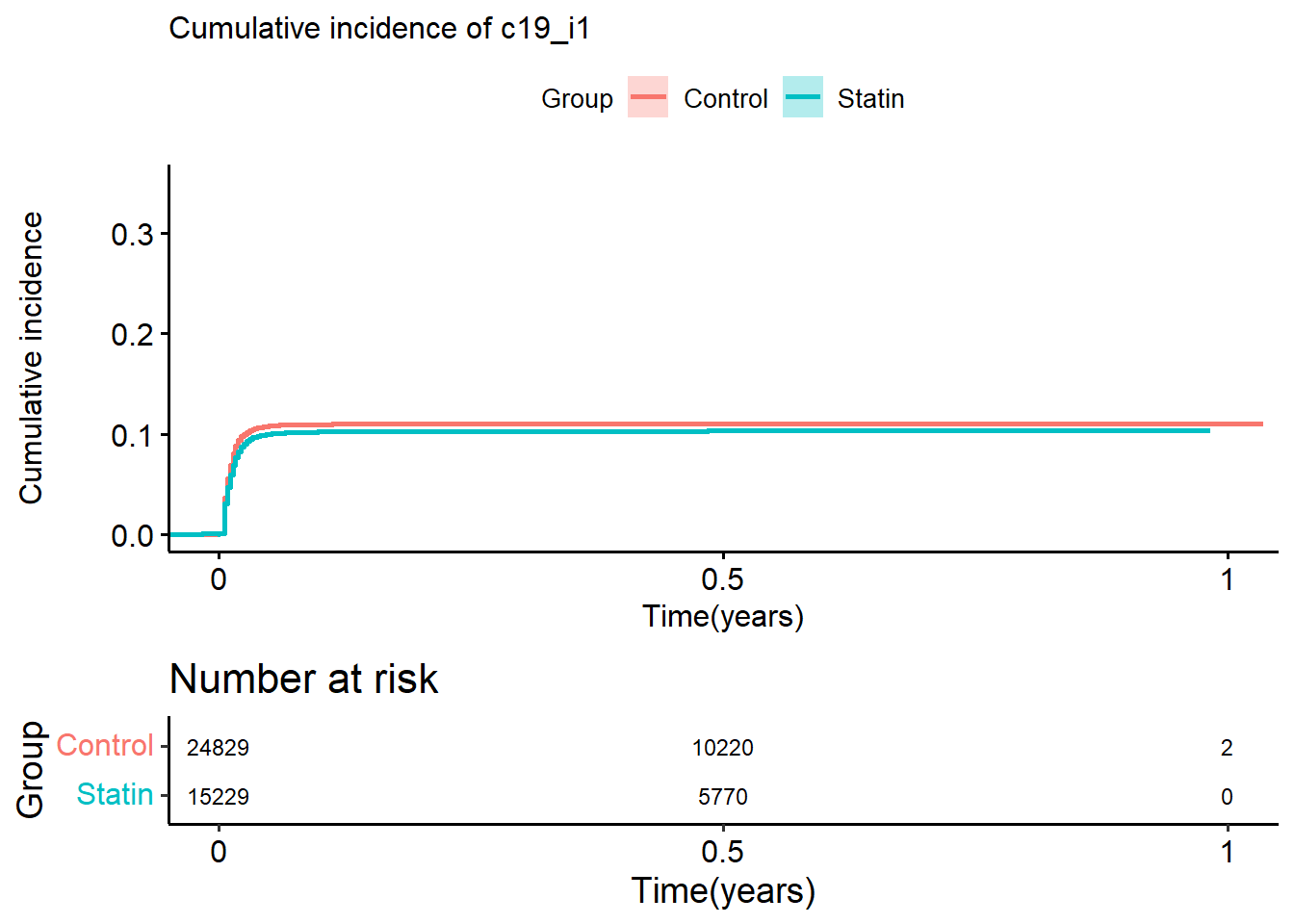

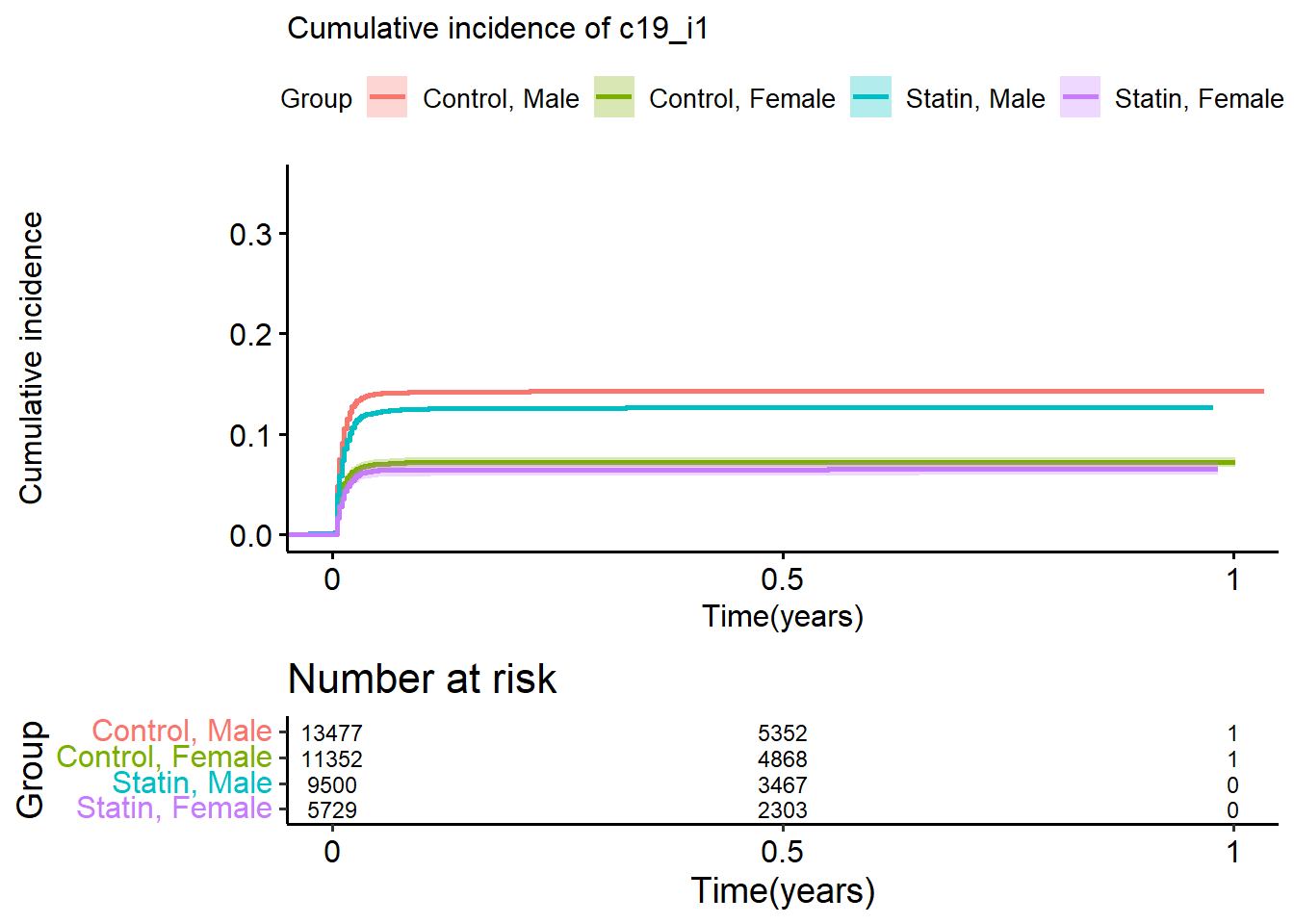


Cumulative incidence of COVID-19 ICU admission


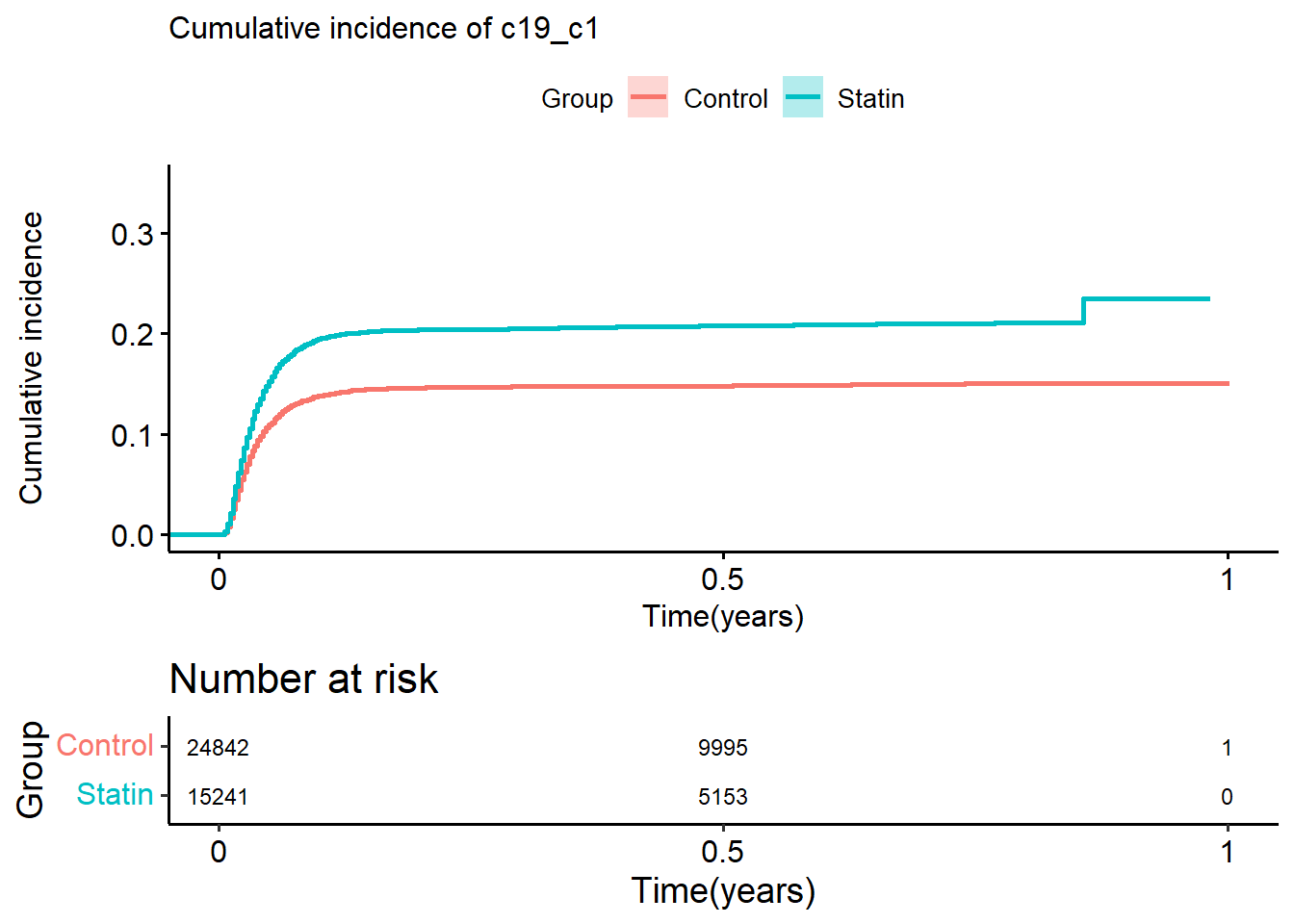

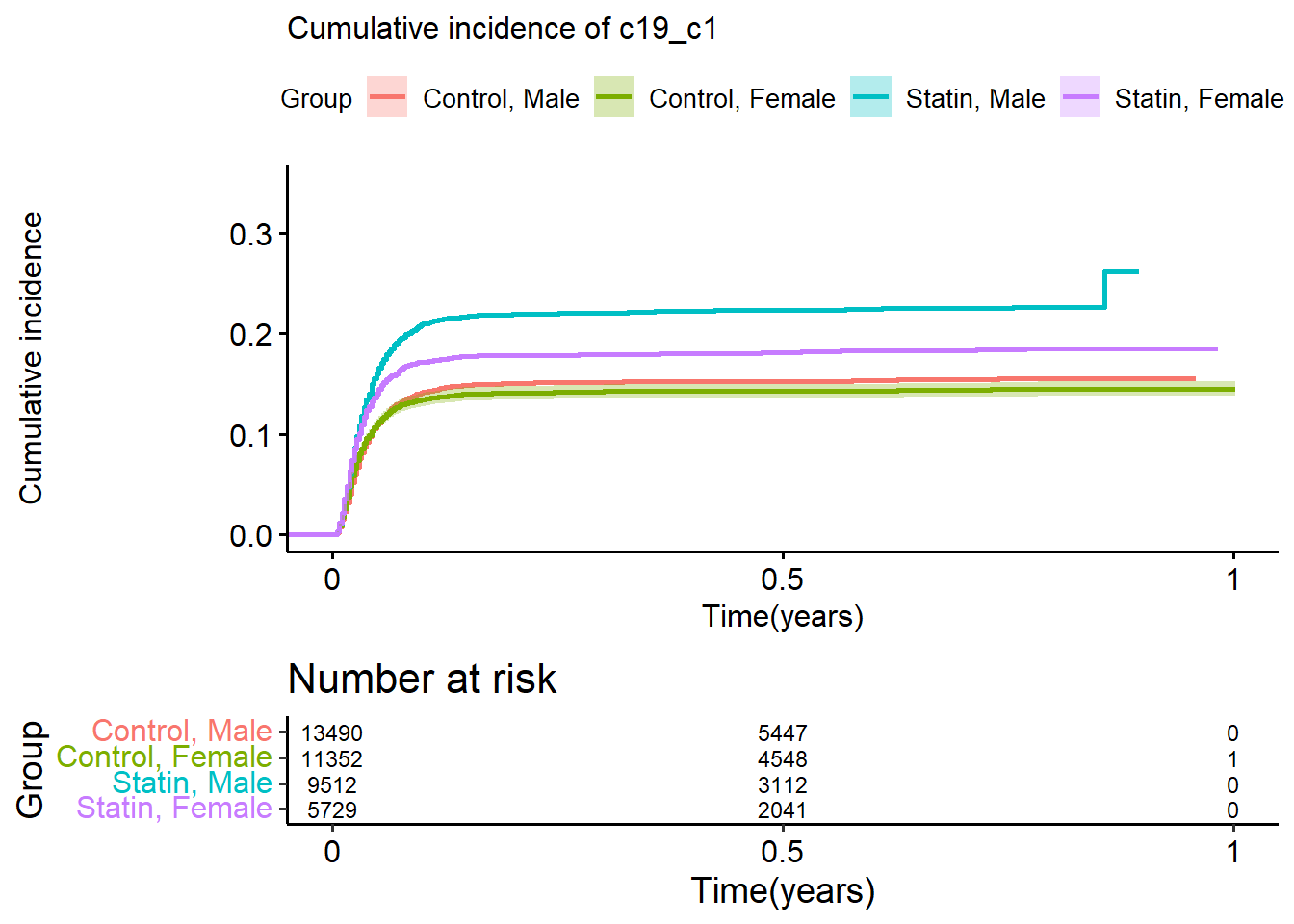


Cumulative incidence of COVID-19 death
